# LINC00115 promotes chemoresistant breast cancer stem-like cell stemness and metastasis through SETDB1/PLK3/HIF1α signaling

**DOI:** 10.1186/s12943-024-01975-3

**Published:** 2024-03-22

**Authors:** Fei Luo, Mingda Zhang, Bowen Sun, Chenxin Xu, Yi Yang, Yingwen Zhang, Shanshan Li, Guoyu Chen, Ceshi Chen, Yanxin Li, Haizhong Feng

**Affiliations:** 1 State Key Laboratory of Systems Medicine for Cancer, Renji-Med X Clinical Stem Cell Research Center, Ren Ji Hospital, Shanghai Cancer Institute, Shanghai Jiao Tong University School of Medicine, Shanghai, 200127 China; 2 Pediatric Translational Medicine Institute, Department of Hematology & Oncology, Committee Key Laboratory of Pediatric Hematology & Oncology, Shanghai Children’s Medical Center, Shanghai Jiao Tong University School of Medicine, National Health Committee Key Laboratory of Pediatric Hematology & Oncology, Shanghai, 200127 China; 3 Academy of Biomedical Engineering, the Third Affiliated Hospital, Kunming Medical University, Kunming, 650500 China

**Keywords:** Cancer stem-like cells, Triple-negative breast cancer, Therapeutic resistance, LINC00115, Lysine methylation, HIF1α

## Abstract

**Background:**

Cancer stem-like cell is a key barrier for therapeutic resistance and metastasis in various cancers, including breast cancer, yet the underlying mechanisms are still elusive. Through a genome-wide lncRNA expression profiling, we identified that LINC00115 is robustly upregulated in chemoresistant breast cancer stem-like cells (BCSCs).

**Methods:**

LncRNA microarray assay was performed to document abundance changes of lncRNAs in paclitaxel (PTX)-resistant MDA-MB-231 BCSC (ALDH^+^) and non-BCSC (ALDH^−^). RNA pull-down and RNA immunoprecipitation (RIP) assays were performed to determine the binding proteins of LINC00115. The clinical significance of the LINC00115 pathway was examined in TNBC metastatic lymph node tissues. The biological function of LINC00115 was investigated through gain- and loss-of-function studies. The molecular mechanism was explored through RNA sequencing, mass spectrometry, and the CRISPR/Cas9-knockout system. The therapeutic potential of LINC00115 was examined through xenograft animal models.

**Results:**

LINC00115 functions as a scaffold lncRNA to link SETDB1 and PLK3, leading to enhanced SETDB1 methylation of PLK3 at both K106 and K200 in drug-resistant BCSC. PLK3 methylation decreases PLK3 phosphorylation of HIF1α and thereby increases HIF1α stability. HIF1α, in turn, upregulates ALKBH5 to reduce m^6^A modification of LINC00115, resulting in attenuated degradation of YTHDF2-dependent m^6^A-modified RNA and enhanced LINC00115 stability. Thus, this positive feedback loop provokes BCSC phenotypes and enhances chemoresistance and metastasis in triple-negative breast cancer. SETDB1 inhibitor TTD-IN with LINC00115 ASO sensitizes PTX-resistant cell response to chemotherapy in a xenograft animal model. Correlative expression of LINC00115, methylation PLK3, SETDB1, and HIF1α are prognostic for clinical triple-negative breast cancers.

**Conclusions:**

Our findings uncover LINC00115 as a critical regulator of BCSC and highlight targeting LINC00115 and SETDB1 as a potential therapeutic strategy for chemotherapeutic resistant breast cancer.

**Supplementary Information:**

The online version contains supplementary material available at 10.1186/s12943-024-01975-3.

## Background

Cancer stem-like cells are a small population of cancer cells that possess infinite proliferative potential and capacity to initiate tumors clonally [1]. Accumulated data demonstrate that cancer stem-like cell is a key barrier for therapy resistance, metastasis, and relapse in various cancers, including breast cancer [2, 3], yet the underlying mechanisms are still elusive.

Long non-coding RNAs (lncRNAs) play a key role in the epigenetic regulation of various cancer characteristics, including proliferation [4], viability [5], and metastasis via interacting with epigenetic modifiers, transcriptional factors/coactivators, or ribonucleoprotein complexes [6–8]. LINC00115 is upregulated in various cancers, including lung adenocarcinoma [9], retinoblastoma [10], prostate cancer [11], ovarian cancer [12], breast cancer [13], and glioma [14]. More importantly, LINC00115 is a prognostic factor for these cancers [9–14]. LINC00115 has been identified as a functional miRNA sponge to regulate miR-200s [14], miR-7 [13], and miR-489-3 [15] in cancer progression. Its upregulation by TGF-β has been observed to mediate the stemness of glioma stem-like cells [14]. Additionally, LINC00115 was shown to enhance stemness and suppress apoptosis in ovarian cancer stem-like cells through the upregulation of SOX9 and the inhibition of the Wnt/β-catenin pathway [12]. However, the role of LINC00115 in therapy-resistant cancer stem-like cells is still unknown.

HIF1α is a common component of pathways involved in the enhancement of breast cancer chemotherapy resistance and metastasis by facilitating breast cancer stem-like cell (BCSC) phenotypes [16]. HIF1α protein stability and activity are mediated by a signaling cascade comprising protein kinases and protein/lipid phosphatases [17]. GSK3β phosphorylates HIF1α within the oxygen-dependent degradation domain, leading to an increased degradation of HIF1α through the proteasomal pathway [18]. PLK3 (Polo Like Kinase 3) can also phosphorylate HIF1α on Ser576 and Ser657 by directly interacting with it, resulting in HIF1α destabilization like that regulated by GSK3β [18, 19]. SETDB1 (SET domain bifurcated histone lysine methyltransferase 1), an H3K9 methyltransferase responsible for methylating lysine 9 on histone H3, has been shown to methylate several nonhistone proteins, such as Akt [20–22]. SETDB1-dependent methylation either augments or impedes the respective kinase activities in the regulation of proliferation, differentiation, and cell fate, as well as repression of cancer [23, 24]. It is interesting but elusive whether SETDB1 is important for HIF1α signaling in breast cancer chemotherapy resistance and metastasis.

In this study, we performed a genome-wide lncRNA expression profiling in paclitaxel-resistant BCSC and identified LINC00115 as a critical regulator. Combining RNA pull-down and mass spectrometry (MS) analyses, we demonstrate that LINC00115 functions as a scaffold lncRNA to regulate BCSC stemness, chemoresistance, and metastasis through SETDB1/PLK3/HIF1α signaling. Our findings uncover a new regulator of therapy-resistant BCSC.

## Methods

### Cell lines and cell culture

MCF10A, MCF7, T47D, BT474, MDA-MB-453, BT549, MDA-MB-468, HCC1806, MDA-MB-231, and human embryonic kidney HEK-293T cell lines were purchased from the Chinese National Infrastructure of Cell Line Resource (Beijing, China). All human cell lines were authenticated using STR DNA fingerprinting at Shanghai Biowing Applied Biotechnology Co., Ltd. (Shanghai, China). MCF7, T47D, BT474, MDA-MB-453, BT549, MDA-MB-468, HCC1806, MDA-MB-231, and HEK-293T were maintained in DMEM supplemented with 10%FBS (Gibco). MCF10A cells were cultured in DMEM/F12 (Invitrogen) supplemented with 20 ng/ml epidermal growth factor, 5% horse serum, 0.5 µg/ml hydrocortisone, 10 µg/ml insulin, and 100 ng/ml cholera toxin. All cell lines were routinely verified to be mycoplasma negative using a mycoplasma detection kit (Sigma-Aldrich).

### Fluorescence-activated cell sorting (FACS)

The ALDH^+^ cell staining was performed by using an ALDEFLUOR assay kit according to the manufacturer’s guidelines (STEMCELL Technologies). Briefly, cells were suspended in ALDEFLUOR assay buffer containing an ALDH substrate, BODIPY-aminoacetaldehyde (BAAA), and incubated for 30 min at 37 °C. Cells treated with diethylaminobenzaldehyde, an ALDH inhibitor, were used as the control. ALDH^+^ cells were sorted by flow cytometer (Beckman Coulter, CytoFLEX). Propidium iodide (PI) staining was used to identify and gate out dead and late apoptotic cells.

### Mammosphere formation and extreme limiting dilution assay (ELDA)

The single-cell suspension was obtained by trypsinization and sieving through a 40-mm sieve. Single cells were plated in ultralow attachment six-well plates (Corning, 3471) at a density of 1 × 10^3^ cells/well and cultured in DMEM/F12 medium plus 20 ng/ml epidermal growth factor (EGF), 20 ng/ml basic fibroblast growth factor (bFGF), 20 µl/ml B27, and 4 mg/ml heparin for 10 days. Then, mammospheres were photographed under an inverted microscope (10× objective, Olympus). For ELDA, cells were seeded into 96-well ultralow attachment plates with sphere medium at densities of 5, 10, 20, 50, and 100 cells/well (12 wells per cell density). After 10 days, each well was examined for the formation of tumor spheres. Stem cell frequency was calculated using extreme limiting dilution analysis (http://bioinf.wehi.edu.au/software/elda/).

### Cell invasion and migration analysis

Cells (5 × 10^4^) suspended in medium without FBS were plated on the upper chamber membranes (8 μm pore size, Corning) coated with or without Matrigel (BD Biosciences). The inserts were incubated in a medium supplemented with 10% FBS for 16 h. To evaluate the invasion and migration ability, non-invasive or non-migrative cells were removed. Invasive and migrative cells were fixed with methanol, stained with crystal violet, and counted under a light microscope.

### RNA pull-down assay

LncRNA was transcribed and amplified in vitro according to the manufacturer’s instructions by using MEGAscript (Ambion, life technologies). Cell nuclear lysates were incubated with biotinylated lncRNA and streptavidin beads for RNA pull-down incubation. In brief, biotinylated RNA was refolded in NEB enzyme buffer with RNase-out (Invitrogen, USA) and then incubated with cell nuclear lysates and streptavidin beads for RNA pull-down incubation. For the pull-down incubations, MDA-MB-231 BCSC lysates were pre-cleared with streptavidin beads and then incubated with biotinylated RNA for 4 h at 4 °C. Beads were collected and washed with RNA binding buffer. RNA-associated proteins were eluted and resolved by SDS/PAGE followed by silver staining (Bio-Rad, USA). Bands were excised and subjected to LC-MS/MS sequencing and data analysis at Jiyun Biotech. Inc (Shanghai, China).

### RNA immunoprecipitation

RNA immunoprecipitation (RIP) experiments were performed using the Megna RIP RNA-binding Protein Immunoprecipitation Kit (Millipore). The anti-YTHDF2 antibody (#71,283, Cell Signaling Technology) was used. The co-precipitated RNAs were detected by qRT-PCR. The gene-specific primers used for detecting LINC00115 are listed in Supplementary Table S2.

### Plasmid construction

LINC00115, SETDB1, PLK3, and HIF1α cDNAs were purchased from GeneChem (Shanghai, China). They were sequenced and subcloned into the pcDNA3.3 or pLVX-Puro vector (Clontech). PLK3 or SETDB1-truncated constructs were generated by PCR using PLK3 or SETDB1-pcDNA3.3 as templates, and then they were inserted into pcDNA3.3. Point mutations were generated using a site-directed mutagenesis kit (Invitrogen) following the manufacturer’s protocol. GST-HIF1α were inserted into a pGEX-4T-1 vector for expression in *E*. coli.

### shRNA-knockdown, sgRNA-knockout, antisense oligonucleotide (ASO), and transfection assays

LINC00115, SETDB1, HIF1α, and YTHDF2 shRNAs were purchased from Genechem (Shanghai, China). LINC00115-ASO purchased from RiboBio (Guangzhou, China). ALKBH5 shRNAs were gifted by Professor Xudong Wu as previously described [25]. Single-guide RNA (sgRNA) sequences of SETDB1 were designed using the online tool from the MIT online tool (http://crispr.mit.edu). Targeted DNAs and packaging plasmids were transfected into the HEK293T cells using the Hieff Trans Liposomal Transfection Reagent (40802ES08, YEASEN) following the manufacturer’s instruction. The supernatants were collected and filtered at 72 h after transfection. Targeted Cells were infected with 5 µg/ml polybrene (Sigma-Aldrich). Infected cells were selected with puromycin after infection. Multiple monoclonal cultures were screened for sgRNA by Western blotting and RT-PCR analyses.

### Immunoprecipitation (IP) and Western blotting (WB) assays

IP and WB assays were performed as previously described [26]. In brief, to detect protein interactions, cells were lysed in IP buffer. These supernatants were immunoprecipitated with the indicated antibodies, slowly shaken on a rotating shaker at 4 °C overnight, and then incubated with Pierce™ Protein A/G Magnetic beads (Invitrogen) at room temperature for 1 h. Then, the binding proteins were eluted and boiled in 1× SDS loading buffer to prepare the samples for immunoblot analysis.

### Antibodies and reagents

Antibodies against SETDB1 (1:1000 for WB, 1:200 for IHC and Co-IP, #2996), PLK3 (1:1000 for WB, 1:200 for Co-IP, #4896), GAPDH (1:1000 for WB, #5174), HIF1α (1:1000 for WB, 1:200 for IHC, #48,085), LDHA (1:1000 for WB, #3582), MDR1 (1:1000 for WB, #12,683), Mono-Methyl Lysine (1:1000 for WB, #14,679), Di-Methyl Lysine (1:1000 for WB, #14,117), Tri-Methyl Lysine (1:1000 for WB, #14,680), methyl-Histone H3 (Lys9) (1:1000 for WB, #13,969), ALKBH5 (1:1000 for WB, 1:100 for RIP, #80,283), YTHDF2 (1:1000 for WB, 1:100 for RIP, #71,283), N6-Methyladenosine (m6A) (3 µg for Me-RIP, #56,593), HA (1:1000 for WB, 1: 200 for Co-IP, #3724), His (1:1000 for WB, 1:200 for Co-IP, #12,698) were purchased from CST (Danvers, MA); an antibody against FLAG (1:1000 for WB, 1:50 for Co-IP, F3165) was from Sigma-Aldrich (US); an antibody against Phospho-Ser/Thr (1:1000 for WB, ab17464) was purchased from Abcam (UK). The secondary antibodies, anti-rabbit IgG, HRP-linked antibody (1:5000, #7074), and anti-mouse IgG, HRP-linked antibody (1:5000, #7076) were purchased from CST (Danvers, MA). PLK3-K106 and -K200 mono-methylation rabbit polyclonal antibodies were raised by Proteintech (US) using the peptide E-K(me)-ILNEIELH and LGNFFITENMEL-K(me) (1:1000 for WB, 1: 50 for IHC). Paclitaxel, CHX, and TDD-IN were from Med Chem Express. MG-132 was from Selleck Chemicals.

### LncRNAs microarray and data analysis

For microarray analysis, RNA purity and integrity were analyzed by Agilent Bioanalyzer 2100 (Agilent). Qualified total RNA was further purified by RNeasy mini kit (QIAGEN) and RNase-free DNase set (QIAGEN). Sample labeling, microarray hybridization, and washing were conducted in accordance with the manufacturer’s standard protocols. Total RNA was transcribed to double-stranded cDNA, and then cRNA was synthesized. Subsequently, 2nd cycle cDNA was synthesized from cRNA. Following fragmentation and biotin labeling, the 2nd cycle cDNA was hybridized onto microarrays. After washing and staining, the arrays were scanned with an Affymetrix Scanner 3000 (Affymetrix). Affymetrix GeneChip Command Console (version 4.0, Affymetrix) software was used to extract the raw data. The Expression Console (version 1.3.1, Affymetrix) software provided RMA normalization for both gene- and exon-level analysis. Additionally, genespring software (version 14.9; Agilent Technologies) was used to complete the fundamental analysis. Differentially expressed lncRNAs were subsequently identified via fold change and p-values calculated by t-tests. The thresholds for up- and downregulated genes were fold-change > 2 and *P* ≤ 0.05.

### RNA-Seq analysis

RNA-Seq and differentially expressed gene analysis were performed as previously described [26]. In brief, total RNAs were extracted and purified according to the RNeasy Plus kit (Qiagen, 74,104). Libraries were prepared using NEB Next® Ultra TM RNA Library Prep Kit for Illumina® (NEB, Beverly, MA, USA) following the manufacturer’s recommendations. The products were sequenced on the HiSeq3000 platform (Illumina, San Diego, CA, USA). The raw FASTQ files were trimmed for adapter sequences using quart. Then HISAT2 version 2.0.5 software was used to map to the hg19 reference genome with default [27] settings. Genes with an adjusted q value < 0.05 and FC > 2 were assigned as differentially expressed.

### RNA extraction and quantitative real-time PCR

Total RNA was isolated from lung tissue and cells using TRIzol reagent (Invitrogen, USA) according to the manufacturer’s instruction. Total RNA from each sample was reverse transcribed to cDNA using the PrimeScript RT kit (Takara, Japan). Quantitative real-time PCR (qPCR) was performed on a Roche Light Cycler 480 System (Roche, Switzerland) using the SYBR Premix Ex Taq II RT-PCR Kit (Takara, Japan) to determine the transcript levels of the target genes. Supplementary Table S2 list the primer sequences. GAPDH was used as the internal reference gene for lung tissues and cells, and the relative expression of the lncRNAs was calculated by the − 2^△△Ct^ method [28].

### MeRIP-qPCR

Total RNAs were extracted with RNAiso Plus (TaKaRa). For meRIP, the procedure was described previously. In brief, purified mRNAs (5 µg) were digested by DNase I (M0303, NEB) and then fragmented into around 200 nt fragments by incubation at 95 °C for 25 s in RNA Fragmentation Reagents (Ambion, AM8740), followed by standard ethanol precipitation and collection. Anti-m^6^A antibody (10 µg antibody for 5 µg mRNAs; Synaptic Systems) was incubated with 40 µl Protein A beads (Sigma, P9424) in IPP buffer (150 mM NaCl, 0.1% NP-40, 10 mM Tris-HCl, pH 7.4) for 2 h at room temperature. The fragmented mRNAs (5 µg) were incubated with the prepared antibody-bead mixture for 4 h at 4 °C. By washing three times, bound RNA was eluted from the beads with 0.5 mg/ml N6-methyladenosine (BERRY & ASSOCIATES, P3732) in IPP buffer. The eluted RNA was extracted by Enol:Chloroform:Isoamylol (pH < 5.0, Solarbio life science, P1025) and then generated to cDNA using 5 x All-In-One RT MasterMix (ABM, G490). The enrichment of m6A was quantified by qPCR. The sequences of qPCR primers are listed in Supplementary Table S2.

### RNA fluorescence in situ hybridization (FISH)

For in situ detection of LINC00115 in breast cancer tissues, FITC-labeled LINC00115 probes, U6 probes, and 18S probes were designed and synthesized by PinpoRNA (China). Cell or tissue FISH assay was performed with a Fluorescence in situ Hybridization Kit (PinpoRNA, China) according to the manufacturer’s instructions. Confocal laser scanning microscopy (Leica, Germany) was used to observe the images.

### Drug sensitivity assays to paclitaxel and TDD-IN

Cells were seeded onto 96-well plates at an initial density of 5 × 10^3^ cells per well and treated with different doses of paclitaxel and TDD-IN. Sensitivity was assayed using CellTiter-Glo (Promega), which measures cellular ATP levels as a surrogate for cell number and growth, according to the manufacturer’s protocol. The solution was diluted 1 part CellTiter-Glo to 2 parts PBS before a 1:1 addition to the volume on the plate. Luminescence was measured using a PerkinElmer Envision.

### Mouse experiments

All animal experiments were conducted under the Institutional Animal Care and Use Committee (IACUC)-approved protocols at Ren Ji Hospital, Shanghai Jiao Tong University School of Medicine in accordance with NIH and institutional guidelines. For the immunodeficiency mouse model, MDA-MB-231 PTX-resistant cells (1 × 10^5^) or their derivatives were suspended in 100 µl of PBS and then injected into the tail vein of nude mice (6 mice for each group). For in vivo drug treatment, 7 days after inoculation, tumor-bearing mice were treated randomly with or without TDD-IN (50 mg/kg), paclitaxel (10 mg/kg), and LINC00115-ASO (10 mg/kg) intraperitoneally every day 21 days after injection, luciferin was injected and the primary/metastatic tumors were detected by BLI with the IVIS 100 (Caliper Life Sciences, Hopkinton, MA, USA). Metastasis in the lungs was detected by bioluminescence imaging (BLI). Animals were treated with isoflurane and then sacrificed with CO_2_ when showing a symptom of cachexia or multiple organ failure. All lobes of the lung were harvested and the number of macroscopic metastatic nodules on the surface of the lung was counted.

### Histology and immunohistochemical staining

Hematoxylin-eosin (H&E) and immunohistochemistry (IHC) stainings were performed on 4-µm formalin-fixed paraffin-embedded tissue sections. The mice’s lung sections were stained with H&E and scanned using a Scanscope XT digital slide scanner (Aperio Technologies). Digital images of lung sections were used to analyze the metastatic burden. IHC for patients’ tissues was performed using anti-SETDB1 (1:100), anti-PLK3K106me1 (1:50), anti-PLK3K200me1 (1:50), and anti-HIF1α (1:100) antibodies. The staining extent score was on a scale of 0–3, corresponding to the percentage of immunoreactive tumor cells (0-10%, 11-25%, 26-75%, and 76-100%, respectively) and the staining intensity (negative, score = 0; weak, score = 1; strong, score = 2; very strong, score = 3). A score ranging from 0 to 3 was calculated by multiplying the staining extent score with the intensity score, resulting in a low (0–1) level or a high (2–3) level value for each specimen. The stained tissues were scored by three individuals blinded to the clinical parameters.

### Human tissue samples

All paraffin-embedded sections of clinical TNBC metastatic lymph nodes were collected at Ren Ji Hospital, Shanghai Jiao Tong University School of Medicine in accordance with a protocol approved by Shanghai Jiao Tong University Institutional Clinical Care and Use Committee of Renji Hospital (Shanghai, China). These patients received chemotherapy, and were with locally advanced or metastatic TNBC progressed after standard chemotherapy including taxol. Informed consent was obtained from all patients. These specimens were examined and diagnosed by independent pathologists. All the research was performed according to the provisions of the Declaration of Helsinki of 1975.

### Statistical analyses

The significance of the data between experimental groups was determined by one-way analysis of variance (ANOVA) with Newman-Keuls post-test or unpaired two-tailed Student’s *t*-test. Pearson Chi-square test or Fisher exact test (two-sided) was used to evaluate IHC score levels between different clinicopathological variable groups. Survival analysis was calculated using the log-rank test and the Kaplan-Meier method. Statistically analyzed data are expressed as the mean ± S.D. or mean ± S.E.M., as indicated. *P* < 0.05 was considered statistically significant. All statistical analyses were performed with GraphPad Prism version 8.3 (GraphPad Software Inc., San Diego, CA, USA).

### Data availability

LncRNAs microarray and RNA-Seq data reported in this study have been deposited with the Gene Expression Omnibus under the accession GEO ID: GSE245145. The data supporting the finding of this study are available within the article and its Supplementary Information files or available from the corresponding author on reasonable request.

## Results

### LINC00115 is robustly upregulated in paclitaxel-resistant breast cancer stem cells

**Fig. 1:**
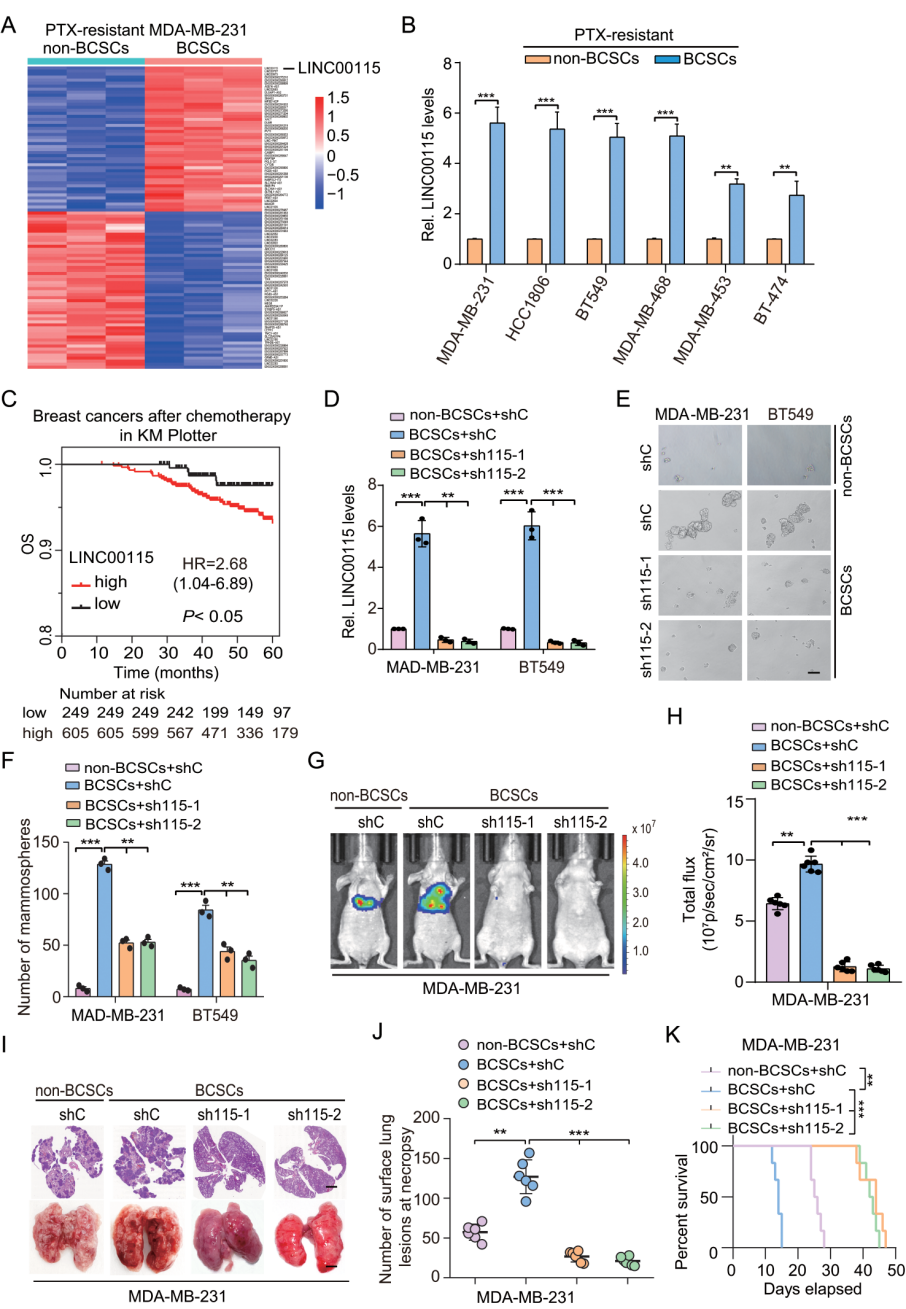
LINC00115 is significantly upregulated in paclitaxel-resistant breast cancer stem-like cells. **A** A heatmap of lncRNAs differentially expressed in paclitaxel-resistant (PTX_R) MDA-MB-231 BCSCs and non-BCSCs. The top 100 lncRNAs with significant differences are shown in the heatmap. **B** QRT-PCR of LINC00115 expression in non-BCSCs and BCSCs from various PTX-resistant breast cancer cells. **C** High LINC00115 expression correlates with reduced overall survival (OS) in breast cancer specimens after endocrine and chemotherapy in the Kaplan-Meier plotter dataset. The cutoff value used in the analysis is -3.32. **D** QRT-PCR of *LINC00115* knockdown (KD) using two different *LINC00115* shRNAs (sh115-1 and sh115-2) or a control shRNA (shC) in PTX-resistant BCSCs and non-BCSCs.E. Representative images of *LINC00115* KD on mammospheres formation of LINC00115-knockdown BCSCs derived from MDA-MB-231/PTX_R and BT549/PTX_R cells. Scale bars, 12.5 μm. **F** Quantification of mammospheres formation in (**E**). **G** Representative bioluminescence images of *LINC00115* KD-decreased tumor metastasis (*n* = 6). Mice were imaged at 14 days after tail vein injection. **H** Quantification of the bioluminescence activity in (**G**). **I** Representative hematoxylin and eosin (**H** & **E**) staining (upper panel) and bright-field imaging (lower panel) of the lungs in (**G**). Scale bars: 100 μm. **J** The number of macroscopic lesions on the lung surfaces in (**G**) was quantified at necropsy. **K** Kaplan-Meier survival of animals with indicated MDA-MB-231 BCSC tumors (*n* = 6).Data are representative of three independent experiments with similar results. Error bars, ± SEM. **P < 0.01, ***P < 0.001, by paired two-tailed *t*-test or log-rank test

To determine the roles of lncRNAs in regulating chemoresistant breast cancer stem cell properties, we generated paclitaxel (PTX)-resistant MDA-MB-231 BCSC (ALDH^+^) and non-BCSC (ALDH^−^) using fluorescence-activated Cell Sorting (FACS) (Supplementary Fig. 1a, b) as previously described [29] and then lncRNA RNA-sequencing was performed. As shown in Fig. 1a and 138 lncRNAs were identified to be robustly upregulated in PTX-resistant MDA-MB-231 BCSC compared to non-BCSC (fold change > 2, *P* < 0.05) and LINC00115 is one of the top lncRNAs. We further performed qRT-PCR analysis and validated that LINC00115 is upregulated in various PTX-resistant BCSC (Fig. 1b). Compared to the human normal breast cell line MCF-10 A, LINC00115 was highly expressed in all examined breast cancer cells (Supplementary Fig. 1c). In addition, the expression levels of LINC00115 were higher in the metastatic breast cancer cell lines (BT549, MDA-MB-468, HCC1806, and MDA-MB-231) than those in non-metastatic breast cancer cell lines (MCF7, T47D, BT474, and MDA-MB-453) (Supplementary Fig. 1c). LINC00115 levels were also higher in mammospheres than those in adherent cells (Supplementary Fig. 1d). Importantly, high LINC00115 expression significantly correlates with a poor overall survival (OS) in breast cancer specimens after endocrine and chemotherapy in the Kaplan-Meier plotter dataset (https://kmplot.com/analysis) (Fig. 1c).

Next, to explore the function of LINC00115 in PTX-resistant BCSC, we knocked down LINC00115 with two different shRNAs, sh115-1 and sh115-2. As shown in Fig. 1d, f and Supplementary Fig. 1e, f, LINC00115 knockdown (KD) markedly reduced mammosphere formation, cell invasion, and migration in MDA-MB-231 and BT549 PTX-resistant BCSC. In contrast, LINC00115 overexpression markedly increased mammosphere formation in MDA-MB-231 and BT549 PTX-resistant non-BCSC (Supplementary Fig. 1g-i). Remarkably, LINC00115 KD significantly decreased MDA-MB-231 BCSC xenograft tumor growth (Fig. 1g, h) and the formation of metastatic lung nodules (Fig. 1i, j) and prolonged animal survival (Fig. 1k). All these findings indicate that an elevated expression of LINC00115 is linked to chemotherapy resistance caused by BCSC and facilitates breast cancer lung metastasis.

### LINC00115 activates the HIF1α pathway and thereby enhances BCSC properties

**Fig. 2:**
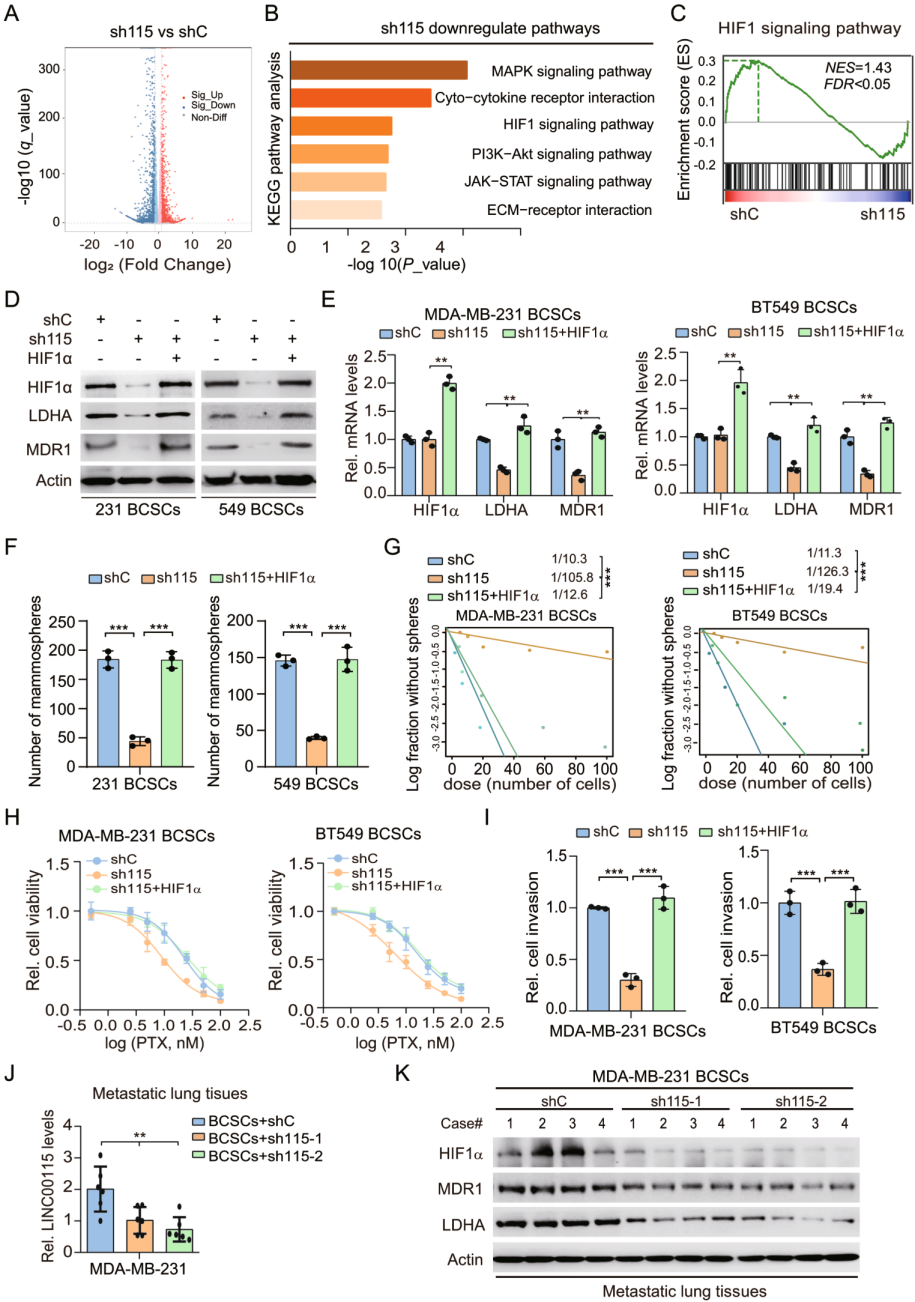
LINC00115 activates the HIF1α pathway and thereby enhances BCSC properties. **A** A volcano plot of differentially expressed genes in LINC00115 KD MDA-MB-231 BCSCs (derived from MDA-MB-231/PTX_R cells) with LINC00115 shRNA-2 (sh115-2). The red dots indicate the significantly upregulated genes and the blue dots indicate the significantly downregulated genes. **B** Kyoto Encyclopedia of Genes and Genomes (KEGG) enrichment analysis of downregulated pathways by LINC00115 KD. **C** GSEA analyses show that LINC00115 KD downregulates the HIF1 signaling pathway. NES, normalized enrichment score. **D** Western blotting (WB) of effects of LINC00115 KD on HIF1 signaling inactivation in MDA-MB-231 and BT549 BCSCs. **E** QRT-PCR of expression of HIF1α, LDHA, and MDR1. **F** Ectopic expression of HIF1α rescues mammosphere formation inhibited by LINC00115 KD. **G** Limiting dilution mammosphere-forming assays were determined and calculated in cells from panel (**D**). **H** Cell viability analysis. BCSCs derived from MDA-MB-231/PTX_R and BT549/PTX_R cells from **D** were treated with paclitaxel for 72 h. Cell viability was determined with Sulforhodamine B colorimetric (SRB) assay. **I** Cells generated in D were subjected to transwell assays and relative invasion numbers were calculated. **J** QRT-PCR of effects of *LINC00115* depletion on LINC00115 levels in metastatic lung tissues from Fig. 1G. **K** WB of effects of *LINC00115* depletion on HIF1α, LDHA, and MDR1 levels in metastatic lung tissues from Fig. 1G. Data are representative of three independent experiments with similar results. Error bars, ± SEM. **P < 0.01, and ***P < 0.001, by paired two-tailed *t*-test

To determine the mechanism by which LINC00115 mediates BCSC properties, we performed RNA-sequencing analysis and identified 1499 genes whose expression was dramatically downregulated by LINC00115 KD in MDA-MB-231 PTX-resistant BCSC (Fig. 2a). Through KEGG pathway mapping, we found1499 genes were highly enriched in various classical cancer-related signaling pathways, and identified the hypoxia-inducible factor 1 (HIF1) signaling pathway, which was associated with BCSC (Fig. 2b). Similarly, gene set enrichment analysis (GSEA) also revealed that the HIF1 signaling pathway was significantly altered by LINC00115 KD (Fig. 2c).

Since the HIF1 signaling is critical for BCSC properties, chemoresistance, and recurrence [30, 31], we focused on this pathway. LINC00115 KD significantly downregulated the protein levels of HIF1α protein levels as well as the protein and mRNA levels of its targets LDHA and multidrug resistance 1 (MDR1), which have been implicated in BCSC and resistance to chemotherapy [32]. Conversely, re-expression of HIF1α substantially rescued LINC00115 KD-reduced expression of LDHA and MDR1 at both the protein and mRNA levels (Fig. 2d and e). Consistently, re-expressing HIF1α reversed LINC00115 KD-reduced mammosphere formation, PTX therapy cell viability, and cell invasion in MDA-MB-231 and BT549 BCSC (Fig. 2f and i). Moreover, LINC00115 KD markedly decreased the levels of HIF1α, LDHA, and MDR1 in metastatic lung tissues (Fig. 2j and k). Our findings elucidate that LINC00115 activates HIF1 signaling to enhance BCSC properties and thereby promotes breast cancer chemoresistance and metastasis.

### LINC00115 links SETDB1 and PLK3 to regulate HIF1α expression

**Fig. 3:**
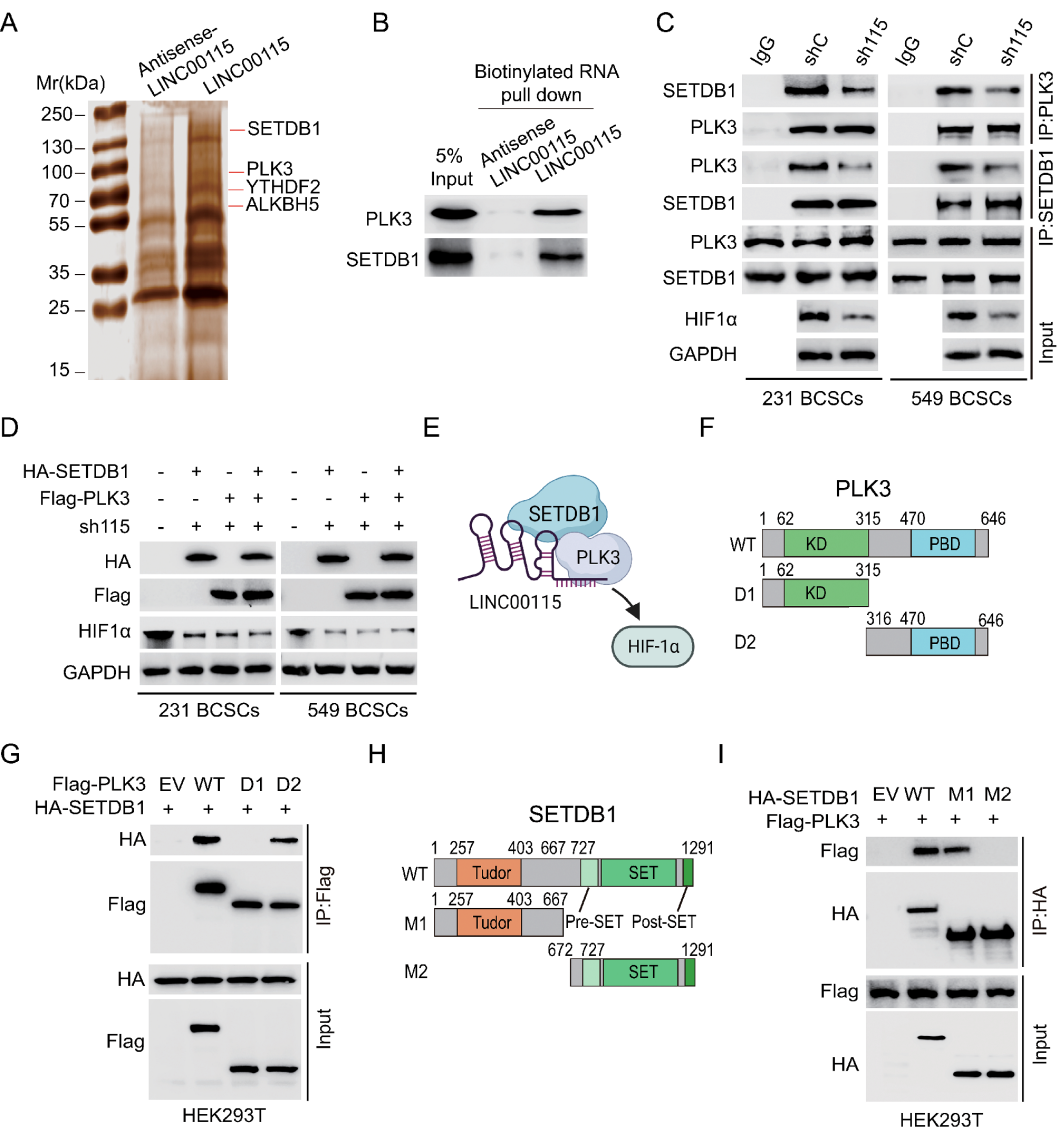
LINC00115 links SETDB1 and PLK3 to regulate HIF1α expression. **A** Representative image of silver-stained PAGE gels showing separated proteins that were pulled down using biotin-labeled LINC00115. LINC00115 and anti- LINC00115 RNA were biotinylated by in vitro transcription, refolded, and incubated with MDA-MB-231 BCSCs total cell lysates. **B** WB of the proteins from antisense LINC00115 and LINC00115 pull-down assays. **C** Effects of LINC00115 KD on SETDB1 associated with PLK3 and HIF1α expression in MDA-MB-231 and B549 BCSCs. **D** Effects of LINC00115 KD on HIF1α expression with or without ectopic expression of HA-SETDB1 or/and Flag-PLK3 in MDA-MB-231 and B549 BCSCs. **E** A schematic model of LINC00115 functions in metastasis. LINC00115 may function as a scaffold lncRNA to recruit SETDB1 and PLK3 to activate the HIF1α signaling pathway. **F** Schematics of PLK3 full length (WT, 1-646aa), D1 (N-terminal, 1-315aa, contain Kinase domain (KD) mutant, and D2 [C-terminal, 316-646aa, contain polo box domain (PBD)] mutant plasmids. **G** Immunoblot analysis (IB) detection of SETDB1 interaction with PLK3 was immunoprecipitated with anti-Flag magnetic beads in HEK-293T cells transfected with the HA-SETDB1 and Flag-PLK3 constructs. **H** Schematics of SETDB1 full length (WT, 1-1291aa), M1 (N-terminal, 1-667aa, contain Tudor domain) mutant, and M2 (C-terminal, 672-1291aa, contain SET domain) mutant plasmids. **I**. IB of SETDB1 interaction with PLK3 and PLK3 methylation was immunoprecipitated with anti-HA magnetic beads in HEK-293T cells transfected with the Flag-PLK3 and HA-SETDB1 constructs

To further investigate the mechanism of LINC00115-mediated BCSC properties, we employed an unbiased approach to identify intracellular LINC00115-binding factors. Biotinylated LINC00115 or antisense LINC00115 RNA (as a negative control) was incubated with total protein extracts from MDA-MB-231 PTX-resistant BCSC and then RNA pulldown analysis was performed using streptavidin beads (Fig. 3a). After mass spectrometry analysis of LINC00115-binding proteins, we identified a range of proteins that possess functions related to post-translational modification, including SET domain bifurcated histone lysine methyltransferase 1 (SETDB1) and Polo-like kinase 3 (PLK3). In addition, we also found proteins that exhibit the capability of RNA m^6^A methylation modification, namely alkB homologue 5 (ALKBH5) and YTH domain proteins YTHDF2 (Fig. 3a and Supplementary Table 1). These binding proteins exhibit a significant correlation with the advancement of breast cancer and other malignant tumors [33–35].

PLK3 has been shown to directly phosphorylate HIF1α and regulate HIF1α stability [19]. Thus, we picked out PLK3 for further study. We performed RNA pull-down analysis and validated that PLK3 bound to LINC00115 (Fig. 3b). We also revealed that SETDB1 bound to LINC00115 (Fig. 3b). LINC00115 KD decreased LINC00115 association with SETDB1 and PLK3 in MDA-MB-231 and BT549 PTX-resistant BCSC (Fig. 3c). Importantly, we found that overexpression of PLK3 and/or SETDB1 did not rescue LINC00115 KD-reduced HIF1α expression (Fig. 3d), suggesting that LINC00115 may link PLK3 association with SETDB1 in regulating HIF1α expression (Fig. 3e).

PLK3 is comprised of a conserved N-terminal serine/threonine kinase domain and a comparatively less conserved C-terminal substrate-binding domain, referred to as the Polo box domain (PBD) (Fig. 3f). To gain a deeper understanding of the molecular mechanism by which LINC00115 recruits the SETDB1/PLK3 complex to activate the HIF1 signaling pathway, we constructed two truncated PLK3 mutants D1 and D2 (Fig. 3f). As shown in Fig. 3g, the mutant D2, but not D1, bound to SETDB1, suggesting that the C-terminus of PLK3 interacts with SETDB1. We further constructed two truncated SETDB1 mutants M1 and M2 (Fig. 3h) and found that the N-terminus of SETDB1 is required for the association of SETDB1 and PLK3 (Fig. 3i). These data indicate that LINC00115 links SETDB1 and PLK3 to regulate HIF1α expression in PTX-resistant BCSC.

### LINC00115 facilitates SETDB1 methylation of PLK3

**Fig. 4:**
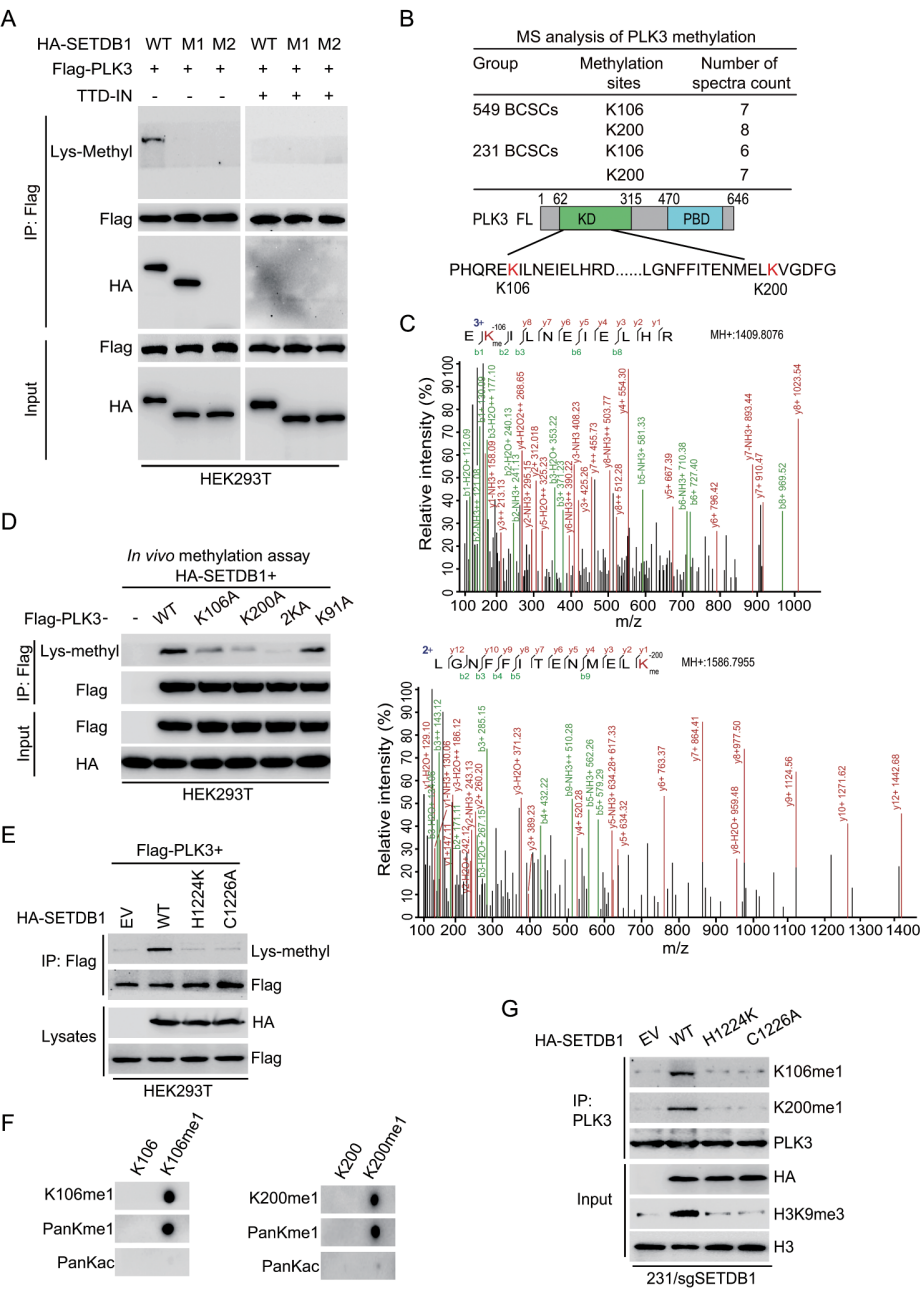
LINC00115 facilitates SETDB1 methylation of PLK3. **A** immunoprecipitation (IP) and IB analysis of PLK3 methylation in HEK293T cells treated with/without SETDB1 inhibitor TTD-IN (25 µM) for 12 h. **B** Mass spectrometry (MS) analysis showing potential methylation sites in PLK3 in BT549 and MDA-MB-231 BCSCs. A schematic representation of its amino acid sequence with all lysine (K) residues was highlighted in red. PB, Polo box domain. **C** LC-MS/MS spectrum of two tryptic peptides with a monomethylated residue at K106 and K200, carrying a mass of + 14.016 Da, respectively. **D** In vivo methylation assay of PLK3 using a pan anti-Lys-methyl antibody. Flag-tagged PLK3 WT or mutants were immunoprecipitated with anti-Flag magnetic beads from HEK293T cells co-expressed with HA-SETDB1. **E** Effects of methyltransferase activity-deficient mutants H1224K and C1226A of SETDB1 on PLK3 methylation in HEK293T cells. **F** Dot blot of PLK3-K106 or -K200 mono-methylation antibody using K106 or K200 unmodified (K106 or K200-free), and K106 or K200 monomethylated (K106 or K200-me1) peptides. **G** Effects of SETDB1 on PLK3 K106 and K200 methylation in MDA-MB-231/sgSETDB1 BCSCs transfected with SETDB1 sgRNA-resistant HA-SETDB1^WT^, SETDB1^H1224K^, or SETDB1^C1226A^ mutant

SETDB1 is a prominent member of the Suppressor of Variegation 3–9 (SUV39)-related protein lysine methyltransferases (PKMTs) and is widely expressed in human tissues [36]. SETDB1 methylates Histone 3 lysine 9 (H3K9) residues and promotes chromatin compaction, exerting negative regulation on gene expression [37]. SETDB1 plays an oncogenic role in various human cancers, specifically related to breast cancer endocrine therapy resistance [33]. To determine whether SETDB1 methylates PLK3, we first performed an immunoprecipitation analysis. As shown in Fig. 4a, ectopic expression of the wildtype (WT), but not the mutants M1 and M2, SETDB1 methylated PLK3. The treatment of TTD-IN, a selective small molecular SETDB1 tandem tudor domain endogenous binder competitive inhibitor [38], blocked SETDB1 methylation of PLK3 (Fig. 4a). LINC00115 KD markedly decreased SETDB1 methylation of PLK3 in MDA-MB-231 and BT549 cells (Supplementary Fig. 2a). In addition, LINC00115 KD reduced PLK3 phosphorylation (p-PLK3) (Supplementary Fig. 2a), suggesting that SETDB1 methylated PLK3 may be related with PLK3 phosphorylation. Moreover, the outcome that LINC00115 KD impaired both SETDB1-methylated PLK3 and PLK3 phosphorylation was not able to be rescued by SETDB1 overexpression (Supplementary Fig. 2a), indicating that the impact of SETDB1 on PLK3 methylation modification is contingent upon the presence of LINC00115.

Next, to identify the putative methylation of lysine (K) residues of PLK3 methylated by SETDB1, we purified SETDB1-associated PLK3 and conducted MS analysis. As represented in Fig. 4b, c, the lysine residues at K106 and K200 of PLK3 were methylated by SETDB1 in both BT549 and MDA-MB-231 PTX-resistant BCSC. These two residues exhibited high conservation across different species (Supplementary Fig. 2b). Methylation analysis in vivo confirmed that mutation of either lysine to arginine (K to A) led to a decrease of SETDB1-induced PLK3 mono-methylation, and notably, double KA mutations abrogated mono-methylation of PLK3 (Fig. 4d). Subsequently, the wild-type variant (SETDB1 WT) or the catalytic inactive mutant of SETDB1 (H1224K and C1226A) [36] were co-transfected with Flag-PLK3 into HEK293T cells. In contrast to SETDB1 WT, the H1224K and C1226A mutations significantly abolished mono-methylation levels of Flag-PLK3 (Fig. 4e).

To further validate the methylation of PLK3 by SETDB1, rabbit polyclonal antibodies specifically recognizing the K106 and K200 mono-methylated PLK3 (Supplementary Fig. 2c) were raised by a commercial vendor. Dot blot analysis was conducted to demonstrate the minimal cross-reactivity of the polyclonal antibody with unmethylated K106 or K200 containing peptides (Fig. 4f). Furthermore, its specificity for PLK3-K106me1 or -K200me1 was verified in MDA-MB-231 cells and a clinical breast cancer specimen (Supplementary Fig. 2d, e). In comparison to re-expression of SETDB1 WT in MDA-MB-231/sgSETDB1 BCSC, re-expression of the H1224K and C1226A mutants did not rescue PLK3-K106me1 and -K200me1 levels as well as the histone H3K9 trimethylation (Fig. 4g). Thus, these data demonstrate that SETDB1 directly methylates PLK3 at the K106 and K200 residues.

### SETDB1 methylation of PLK3 is critical for BCSC properties and metastasis

**Fig. 5:**
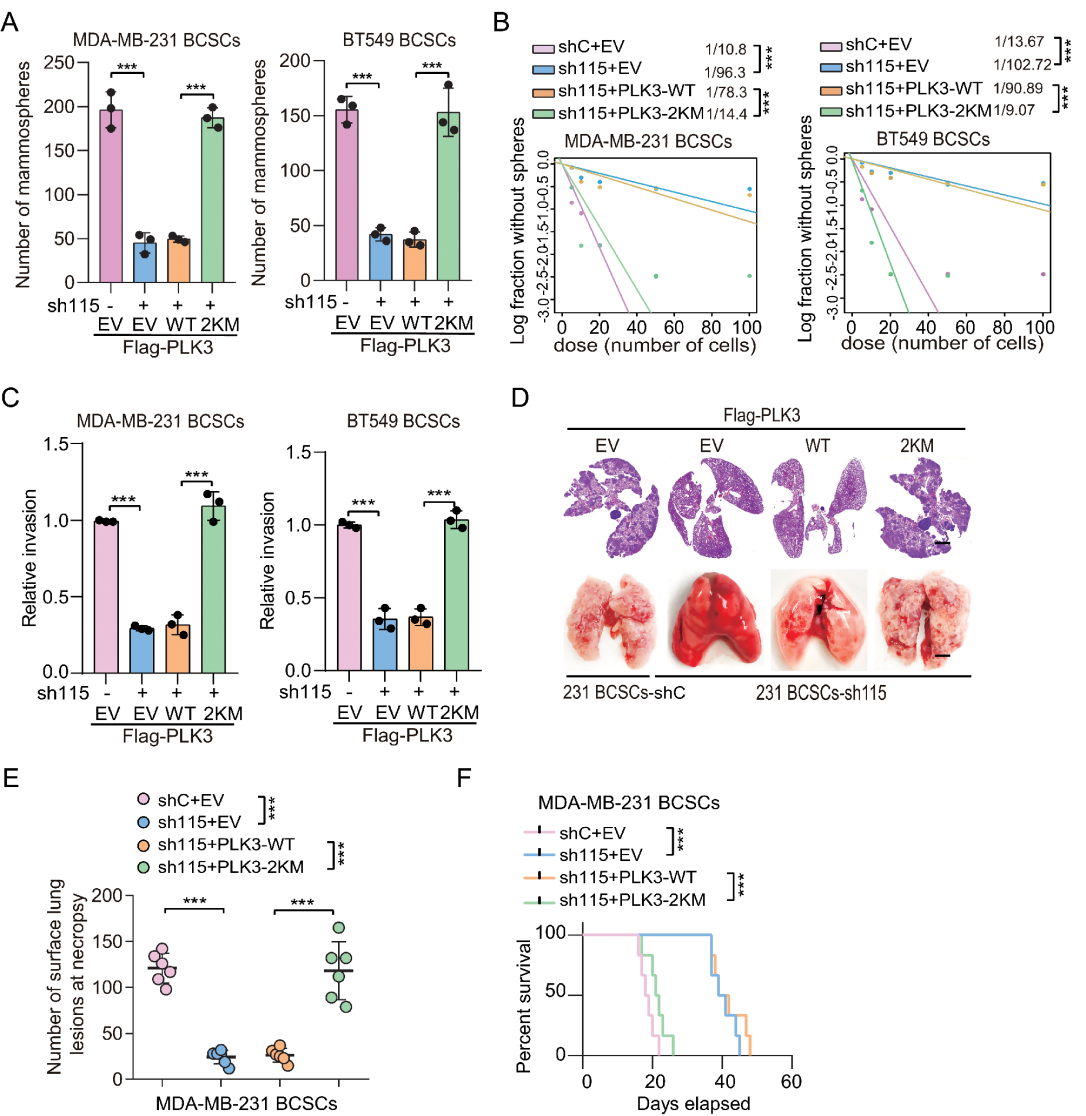
SETDB1 methylation of PLK3 is critical for BCSCs properties and metastasis. **A** Effects of ectopic expression of PLK3 WT or the 2KM mutant on mammospheres formation in MDA-MB-231/sh115 and BT549/sh115 BCSCs. **B** Limiting dilution mammosphere-forming assays of effects of ectopic expression of PLK3 WT or the 2KM mutant. **C**–**F** Effects of ectopic expression of PLK3 WT or the 2KM mutant on cell invasion (**C**), lung metastasis (**D**, **E**), and mouse lifespan (**F**). A cohort of athymic nude mice (*n* = 6 per group) were implanted with 1 × 10^5^ MDA-MB-231 BCSCs which were transfected with either control shRNA (shC) or sh115, with or without ectopic expression of PLK3 WT or PLK3 2KM mutant via the lateral tail vein. After 14 days, the mice were euthanized and the number of macroscopic lesions on the lung surfaces was quantified at necropsy. The cohorts consisted of mice injected with MDA-MB-231 /shC + EV, MDA-MB-231 /sh115 + EV, MDA-MB-231 /sh115 BCSCs with PLK3 WT or the 2KM mutant. Independent experimental groups under the same conditions were performed to calculate the animal survival (*n* = 6). Scale bar, 100 μm. Data are representative of three independent experiments with similar results. Error bars, ± SEM. ***P < 0.001, by paired two-tail *t*-test or log-rank analysis

To investigate the functions of SETDB1-dependent PLK3 methylation in the modulation of BCSC properties and metastasis, we first re-expressed Flag-PLK3 WT and 2KM mutant (the methylation-mimetic PLK3 2KM mutant) in MDA-MB-231 BCSC with or without LINC00115 KD. As shown in Fig. 5a and c, LINC00115 KD led to a significant decrease in mammosphere formation and cell invasion. However, the ectopic expression of the PLK3 2KM mutant, but not the WT, rescued the LINC00115 KD-induced inhibition of mammosphere formation and cell invasion. Consistent with the in vitro observations, the ectopic of PLK3 2KM, but not the WT, reinstated the inhibitory effect of LINC00115 depletion on lung metastasis in vivo (Fig. 5d and e) and decreased LINC00115 depletion-prolonged animal survival (Fig. 5f). These findings provide evidence to support that LINC00115-induced methylation of PLK3 facilitates BCSC properties and metastasis.

### SETDB1 methylation of PLK3 promotes PLK3 phosphorylation and decreases PLK3 activity

**Fig. 6:**
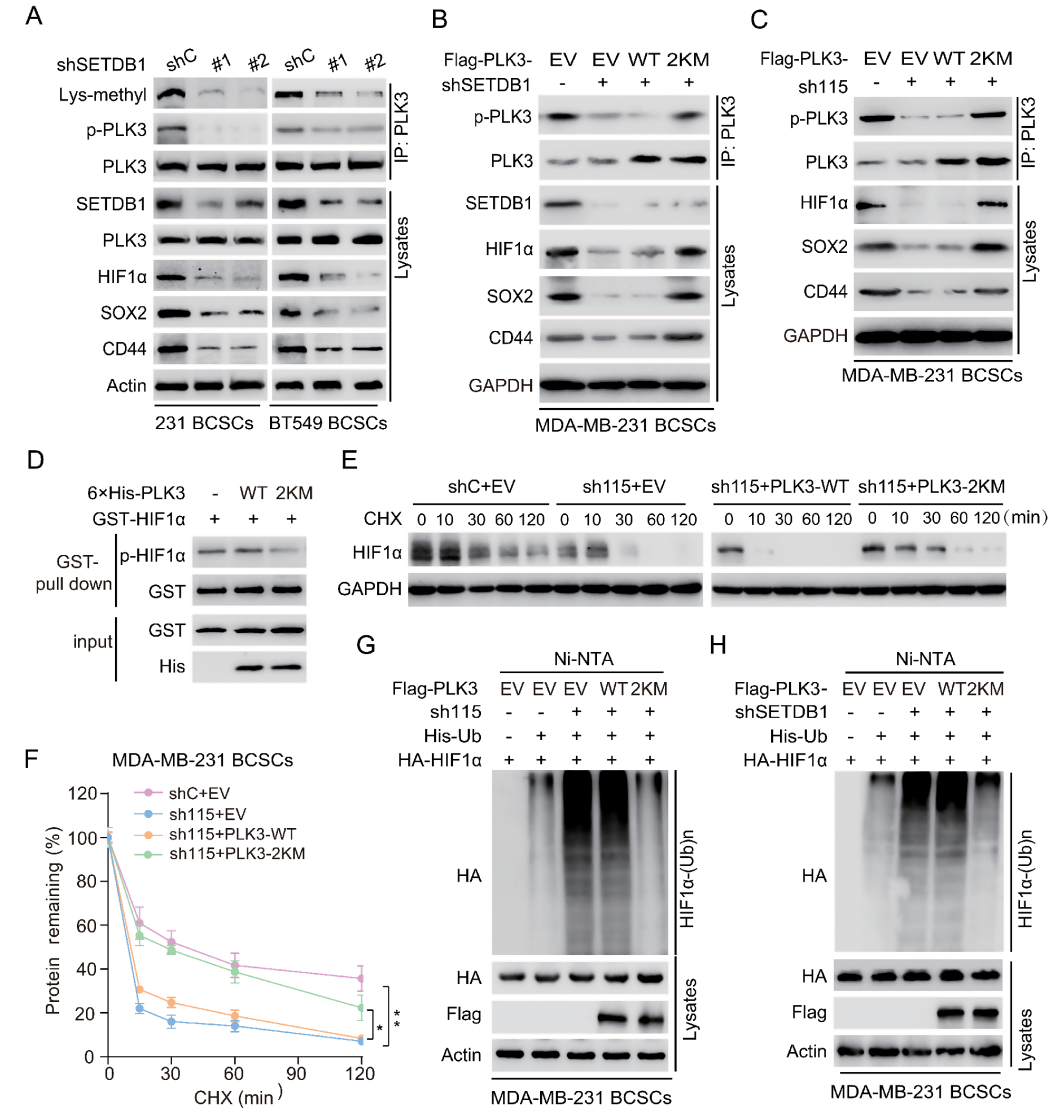
SETDB1 methylation of PLK3 promotes PLK3 phosphorylation and decreases PLK3 activity. **A** IB of the phosphorylation and lysine methylation of PLK3, and also determined the expression of HIF1α, SOX2, and CD44 in control and SETDB1-knockdown MDA-MB-231 and BT549 BCSCs. Lysates were assessed by immunoprecipitation (IP) with anti-PLK3 and immunoblotting with anti-Phospho-(Ser/Thr) and anti-Lys-Mono-Methyl. **B, C** Effects of ectopic expression of PLK3 WT or 2KM (K106M/K200M) mutant on the phosphor-PLK3 and the expression of HIF1α, SOX2, and CD44 in MDA-MB-231 /sh115 (**B**) and MDA-MB-231/shSETDB1 BCSCs (**C**). EV, an empty vector control. Lysates were assessed by immunoprecipitation (IP) with anti-PLK3 and immunoblotting with anti-Phospho-(Ser/Thr). **D** In vitro GST pull-down analysis. Purified GST-HIF1α protein was incubated with cell extracts from MDA-MB-231 BCSCs. **E** and **F** Effects of ectopic expression of PLK3 WT or 2KM mutant on HIF-1α protein degradation in MDA-MB-231/shC or MDA-MB-231/sh115 BCSCs with cycloheximide (CHX, 100 µM) treatment (0, 10, 30, 60, and 120 min). **G** Effects of PLK3 WT or 2KM mutant on HIF-1α ubiquitination in MDA-MB-231/sh115 BCSCs. **H** Effects of ectopic expression of PLK3 WT or 2KM mutant on HIF-1α ubiquitination in MDA-MB-231/shSETDB1 BCSCs.Data are representative of two or three independent experiments with similar results. Error bars, ± SD. **P < 0.01, by paired two-way Student’s *t*-test

To investigate the function of SETDB1 methylation of PLK3, we first assessed the effect of SETDB1 KD on the phosphorylation of PLK3. As shown in Fig. 6a, SETDB1 KD reduced PLK3 methylation and phosphorylation, HIF1α expression, and the expression of BCSC-related proteins SOX2 and CD44 in MDA-MB-231 and BT549 PTX-resistant BCSC. Ectopic expression of the 2KM mutant of PLK3, but not the WT, rescued SETDB1 KD-decreased PLK3 phosphorylation and the expression of HIF1α, SOX2, and CD44 (Fig. 6b). We also revealed that ectopic expression of PLK3 2KM mutant, but not the WT, rescued LINC00115 KD-decreased PLK3 phosphorylation and downstream effector expression (Fig. 6c).

Previous studies have suggested that the phospho-mimicking PLK3 mutant T219D had lower kinase activity compared with PLK3 WT, leading to its ineffectiveness in phosphorylating substrate [39]. Thus, we investigated whether methylation of PLK3 by SETDB1 enhances its phosphorylation and thereby reduces its activity to phosphorylate HIF1α. We carried out glutathione S-transferase (GST) pull-down analysis and found that purified recombinant PLK3 WT phosphorylated HIF1α. However, PLK3 2KM mutant markedly decreased HIF1α phosphorylation compared to the WT (Fig. 6d).

Since HIF1α stability is decreased by its phosphorylation [19], we further investigated the effect of SETDB1 methylation of PLK3 on HIF1α stability. As shown in Fig. 6e and f, LINC00115 KD promoted HIF1α degradation. Compared to the WT mutant, ectopic expression of PLK3 2KM mutant rescued LINC00115 KD-promoted HIF1α degradation. Consistently, ectopic expression of PLK3 2KM mutant, but not the WT, reversed LINC00115 KD or SETDB1 KD-increased HIF1α ubiquitination (Fig. 6g and h). Taken together, these data demonstrate that LINC00115-mediated SETDB1 methylation of PLK3 increases PLK3 phosphorylation and thereby decreases PLK3 activity, resulting in lower HIF1α phosphorylation and higher HIF1α stability.

### HIF1α upregulates ALKBH5 to demethylate LINC00115 m^6^A and enhance LINC00115 stability

**Fig. 7:**
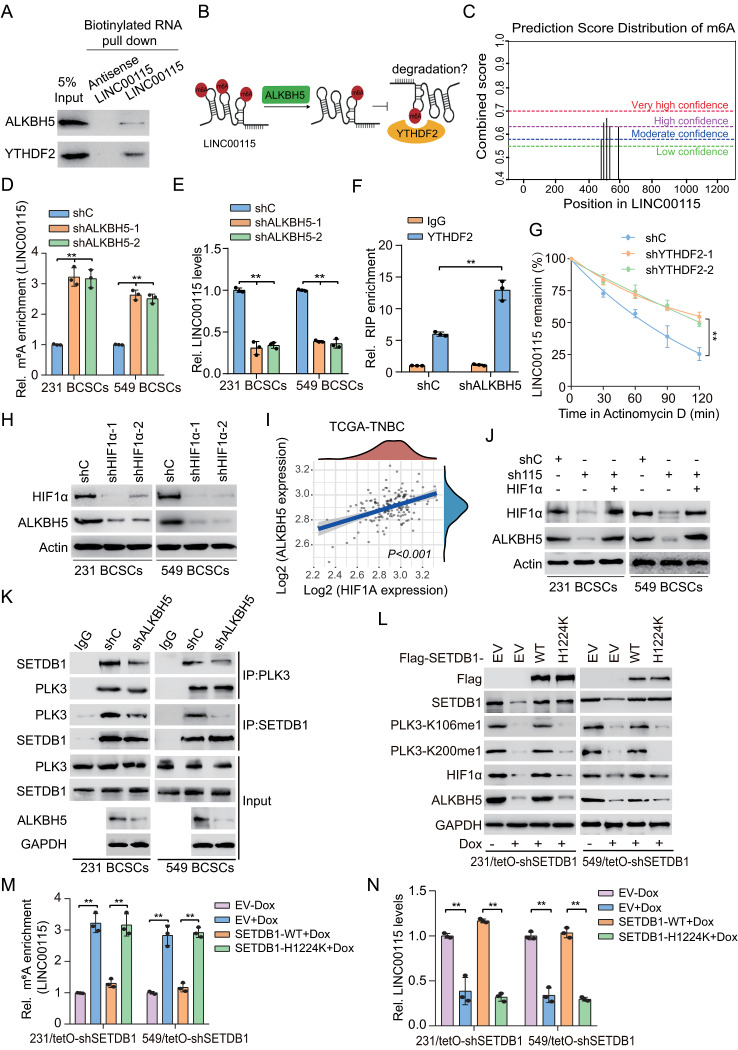
HIFα, in turn, upregulates ALKBH5 to demethylate LINC00115 m^6^A and enhance LINC00115 stability. **A** WB of the proteins from antisense LINC00115 and LINC00115 pull-down assays. LINC00115 combined with ALKBH5 and YTHDF2. **B** A schematic model of LINC00115 stability regulated by ALKBH5 and YTHDF2. The stability of LINC00115 may be regulated through such a mechanism, which ALKBH5 demethylates of LIN00115 m^6^A and decreases YTHDF2-mediated m^6^A LIN00115 degradation. **C** Schematic representation of the position of m^6^A motifs within LINC00115 predicted by SRAMP. **D** m^6^A RNA immunoprecipitation (MeRIP)-qPCR was used to quantify relative LINC00115 m^6^A levels in ALKBH5 KD (shALKBH5-1 and shALKBH5-2) MDA-MB-231 and BT549 BCSCs. **E** QRT-PCR of LINC00115 levels in MDA-MB-231/shALKBH5and BT549/shALKBH5 BCSCs. **F** Interaction between YTHDF2 and LINC00115 was determined by RIP assay in MDA-MB-231 BCSCs with transfection of indicated DNAs. **G** QRT-PCR analysis of LINC00115 stability. MDA-MB-231 BCSCs and MDA-MB-231 YTHDF2 KD (shYTHDF2-1 and shYTHDF2-2) BCSCs were treated with Actinomycin D. **H** HIF1α knockdown impaired ALKBH5 expression in MDA-MB-231 and BT549 BCSCs. **I** Scatter plot of median standard-score-normalized (z-score) expression levels of HIF1α and ALKBH5 across human TNBC metastases after chemotherapy in the TCGA database (*n* = 160). **J** Effects of ectopic expression of HIF1α on ALKBH5 expression in LINC00115 KD MDA-MB-231 and BT549 BCSCs. **K** Effects of ALKBH5 knockdown on SETDB1 association with PLK3. **L** Effects of ectopic expression of Flag-SETDB1 WT or the H1224K mutant on PLK3 methylation and HIF1α and ALKBH5 expression in MDA-MB-231 BCSCs/tetO-shSETDB1 and BT549 BCSCs/tetO-shSETDB1 cells. MDA-MB-231 and BT549 BCSCs with a tetO-shSETDB1 were pretreated with or without Dox. EV, an empty vector control. **M, N** Effects of ectopic expression of SETDB1 WT or H1224K mutant on LINC00115 m^6^A (**M**) and LINC00115 (**N**) levels. Data are representative of two or three independent experiments with similar results. Error bars, ± SD. **P < 0.01, by paired two-way Student’s *t*-test

ALKBH5 (α-ketoglutarate-dependent dioxygenase alkB homolog 5) can remove m^6^A modification from RNA as a m^6^A eraser and thus keeps m^6^A modification in a dynamic balance [40, 41]. YTHDF2, the initial m^6^A reader to be identified, exhibits the ability to recognize and bind to m^6^A-modified RNA, consequently facilitating the process of mRNA or lncRNA decay [42]. Since ALKBH5 and YTHDF2 were identified in the protein complex of LINC00115 (Fig. 3a), we also verified that LINC00115 effectively interacts with ALKBH5 and YTHDF2 (Fig. 7a), thus we further investigate whether LINC00115 stability is regulated by them (Fig. 7b). A few of m^6^A sites in LINC00115 were predicted in a public prediction server (http://www.cuilab.cn/sramp/) [43] (Fig. 7c). We assessed and found that the m^6^A modification levels of LINC00115 exhibited a significant increase upon ALKBH5 KD in PTX-resistant BCSC (Fig. 7d). Conversely, the expression of LINC00115 reduced (Fig. 7e). We further performed RIP analysis and revealed that ALKBH5 KD increased YTHDF2 binding to LINC00115 (Fig. 7f). YTHDF2 KD inhibited LIN00115 degradation (Fig. 7g). These data support that ALKBH5 demethylates LIN00115 m^6^A and decreases YTHDF2-mediated m^6^A LIN00115 decay.

We further found that HIF1α KD decreased ALKBH5 expression (Fig. 7h). Analysis of breast cancer clinical samples from the TCGA database demonstrated a positive association between HIF1α and ALKBH5 mRNA expression (Fig. 7i). Consistently, LINC00115 KD decreased ALKBH5 expression. Ectopic expression of HIF1α rescued LINC00115 KD-inhibited ALKBH5 expression (Fig. 7j). This result indicates that LINC00115-upregulated HIFα, in turn, increases ALKBH5 expression.

Finally, to further investigate whether ALKBH5 feedback regulates SETDB1/PLK3/HIF1 pathway, we performed IP analysis and revealed that ALKBH5 KD impaired the association of SETDB1 and PLK3 (Fig. 7k). In addition, compared to un-induced control, Dox-induced SETDB1 KD decreased SETDB1, PLK3-K106me1, K200me1, HIF1α, and ALKBH5 levels, increased LINC00115 m^6^A modification level, and reduced LINC00115 expression (Fig. 7l and n). Ectopic expression of the SETDB1 WT, but not the H1224K mutant [24], rescued SETDB1 KD-inhibited PLK3 K106me1, K200me1, HIF1α, and ALKBH5 levels, reduced LINC00115 m^6^A levels, and increased LINC00115 expression (Fig. Figure 7l-n). Taken together, our data demonstrate that the SETDB1/PLK3 complex is recruited by LINC00115 to stabilize HIF1α protein, while HIF1α upregulates ALKBH5 to reduce m^6^A modification of LINC00115, resulting in blocking the binding of the m^6^A reader YTHDF2 to stabilize LINC00115. This intricate mechanism forms a positive feedback regulatory loop that actively promotes the progression of breast cancer.

### SETDB1 inhibitor TTD-IN with LINC00115 ASO sensitizes PTX-resistant cell response to chemotherapy

**Fig. 8:**
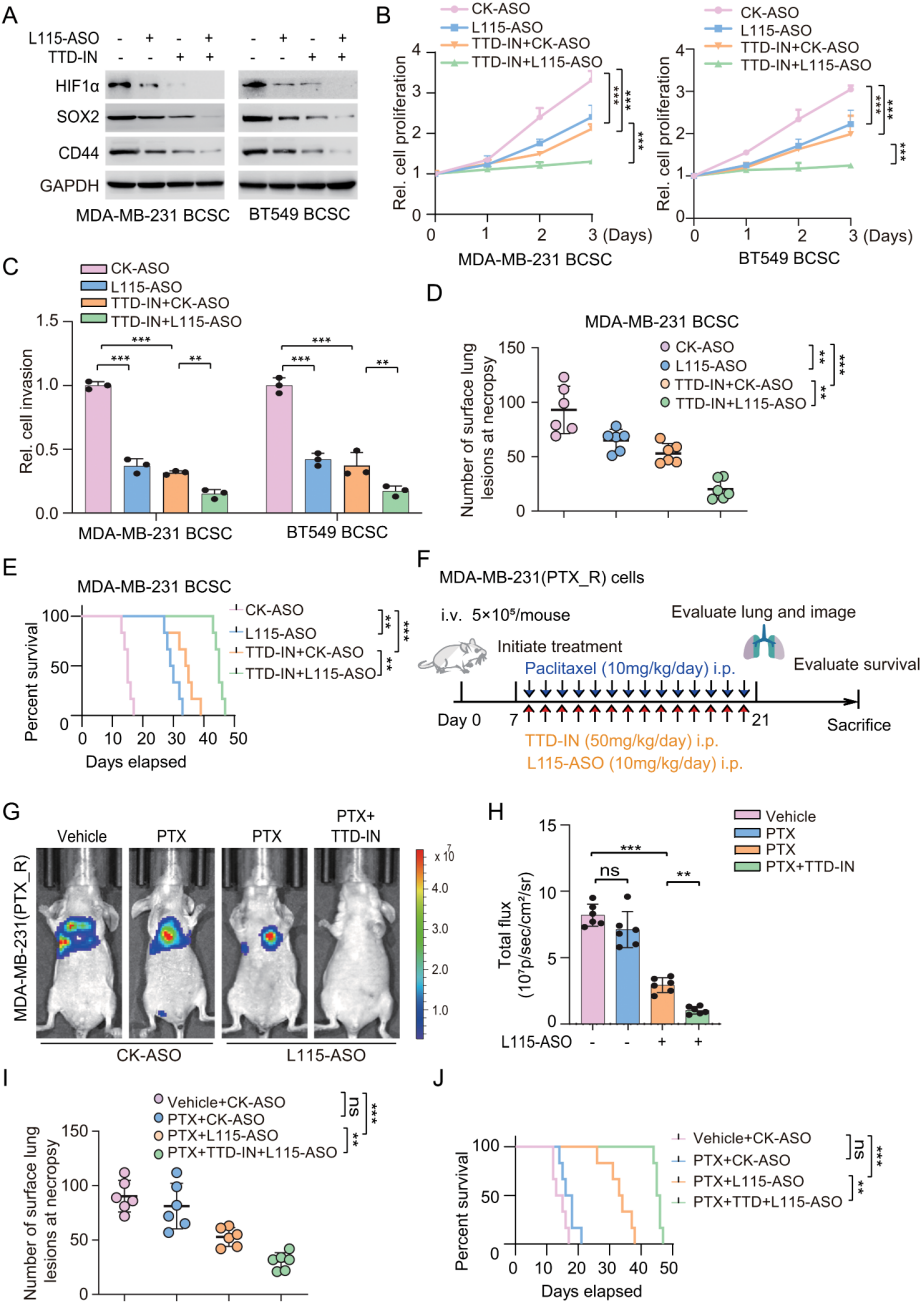
SETDB1 inhibitor TTD-IN with LINC00115 ASO sensitizes PTX-resistant cell response to chemotherapy. **A** Effects of treatment of SETDB1 inhibitor TTD-IN (25 µM) or/and LINC00115 ASO (L115-ASO, 1 µM) on expression of HIF1α, SOX2, and CD44 in MDA-MB-231/PTX_R and BT549/PTX_R cells. Cells were treated with TTD-IN or L115-ASO as indicated. **B**, **C** Cell proliferation (**B**) and invasion (**C**) analyses in MDA-MB-231/PTX_R and BT549/PTX_R cells. **D, E** Effects of treatment of SETDB1 inhibitor TTD-IN (25 µM) or/and L115-ASO on lung metastasis (**D**) and animal survival (**E**). **F** Treatment schedules for the administration of PTX (10 mg/kg, intraperitoneal injection once daily), TTD-IN (50 mg/kg, intraperitoneal injection once daily), or/and L115-ASO (10 mg/kg, intraperitoneal injection once daily) to mice grafted with MDA-MB-231/PTX_R cells (*n* = 6). Control mice received a placebo (vehicle). i.v., intravenous injection. i.p., intraperitoneal injection. **G** Representative bioluminescence images in (**F**) on day 21. **H** Quantification of the bioluminescence activity of MDA-MB-231 tumor xenografts in (**F**). **I** Quantification of the number of surface lung metastasis in panel (**F**). **J** Kaplan-Meier survival analysis of mice with MDA-MB-231 tumor xenografts (*n* = 6). ns, no significance. Data are representative of two or three independent experiments with similar results. Error bars, ± SEM. **P < 0.01, ***P < 0.001, by paired two-way Student’s-test, one-way ANOVA, or log-rank analysis

Inhibitors targeting epigenetic factors have been explored for cancer therapies and have undergone clinical trials, including DNA methyltransferase 1 inhibitors, histone deacetylase inhibitors, and histone methyltransferase inhibitors [44–46]. We also have demonstrated that the selective endogenous binder competitive inhibitor of SETDB1-TTD, TTD-IN [38], significantly inhibited the binding of PLK3 to SETDB1. Considering the critical role of the SETDB1 in contacting and methylating PLK3 and then contributing to drug-resistance and metastasis in LINC00115-driven BCSC, we assessed the effects of TTD-IN treatment combined with LINC00115 antisense oligonucleotides (ASO) on breast cancer metastasis. As shown in Fig. 8a, the treatment of LINC00115-ASO (L115-ASO) or TTD-IN exhibited a modest inhibitory effect on the expression of HIF1α and BCSC marker as well as on cell proliferation and invasion in MDA-MB-231/PTX_R and BT549/PTX_R cells, respectively (Fig. 8a-c). Strikingly, the combined treatment of LINC00115 ASO and TTD-IN demonstrated greater efficacy compared to the individual treatment in both breast cancer cell lines (Fig. 8a-c). Consistent results were also observed in animal models in vivo (Fig. 8d and e).

To furthermore examine the therapeutical potential of targeting epigenetic factors in the treatment of PTX-resistant breast cancer in the foreseeable future, athymic nude mice were intravenously injected with MDA-MB-231/PTX_R cells. After 7 days with inoculation, animals transplanted with MDA-MB-231/PTX_R were treated with either PTX (10 mg/kg, i.p.), TTD-IN (50 mg/kg, i.p.), or/and L115-ASO (10 mg/kg, i.p.) daily for another 14 days. Tumor metastasis was monitored at day 21 post-implantation (Fig. 8f). The group receiving PTX treatment alone did not exhibit a statistically significant inhibitory effect on lung metastasis and survival improvement (Fig. 8g and j). Inhibition of LINC00115 in combination with PTX pronounced reduced lung metastasis and extended survival of animals (Fig. 8g and j). Moreover, the combination treatment of paclitaxel, TTD-IN, and inhibition of LINC00115 further decreased tumor burden, lung metastasis, and significantly improved animal survival (Fig. 8g and j). These findings collectively indicate that targeting LINC00115 and SETDB1 opens a potential therapeutic avenue for therapeutic-resistant breast cancer.

### Correlative expression of LINC00115, methylation PLK3, SETDB1, and HIF1α are prognostic for clinical TNBC

**Fig. 9:**
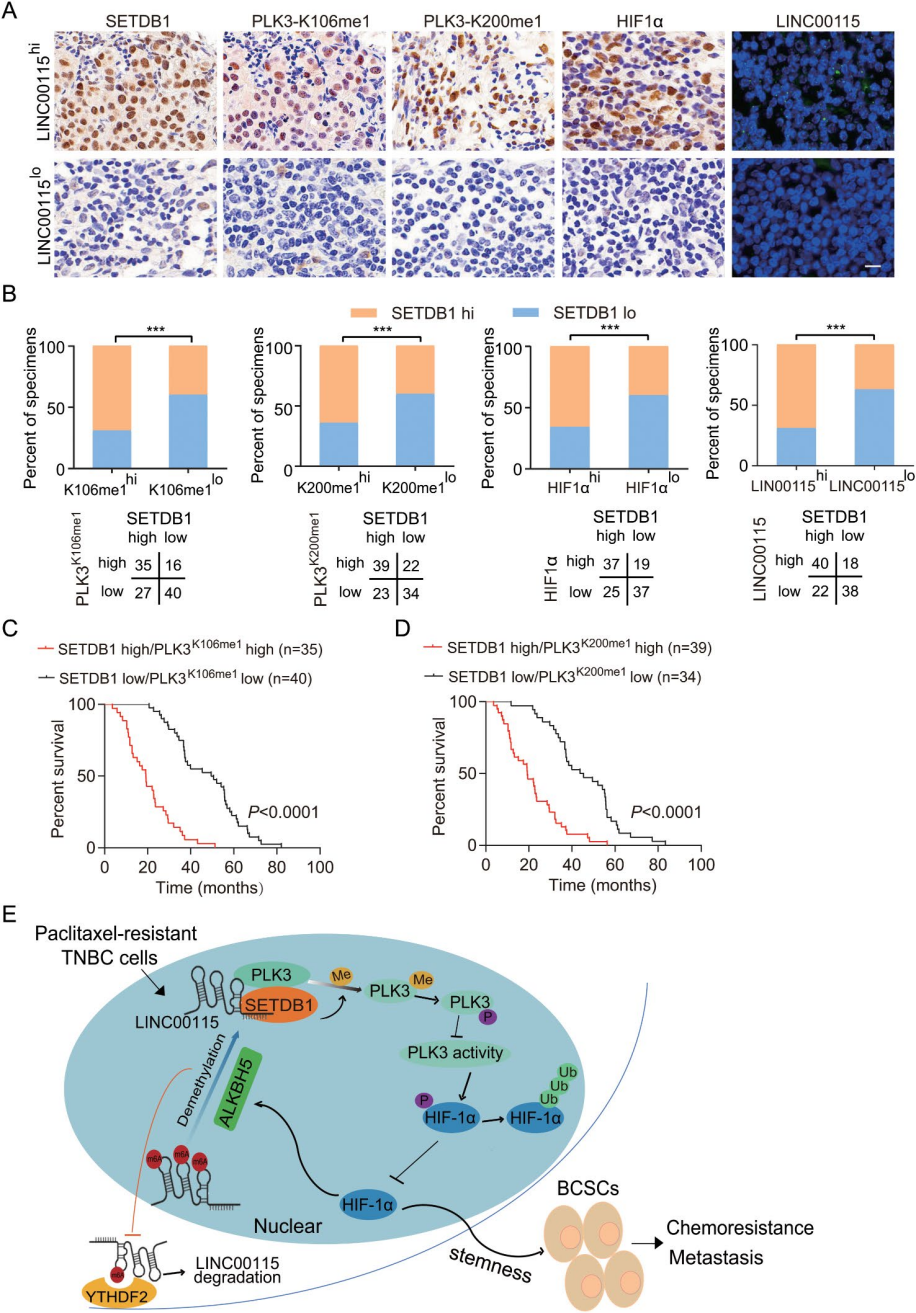
Correlative expression of LINC00115, methylated-PLK3, and SETDB1 are prognostic. **A** Representative images of SETDB1, PLK3-K106me1, PLK3-K200me1, HIF1α, and LINC00115 in 118 clinical TNBC metastatic lymph node tissues. Scale bars, 50 μm. **B** Correlation of expression levels between SETDB1, PLK3K-106me1, PLK3K-200me1, HIF1α, and LINC00115. **C** Prognosis comparison of breast cancer patients with SETDB1/PLK3-K106me1 or -K200me1 ectopic differential expression using Kaplan-Meier survival analysis. **D** A working model of LINC00115 regulating chemoresistance breast cancer cell stemness and metastasis through SETDB1/PLK3/HIF1α signaling. PTX therapy upregulates LINC00115 in BCSCs. LINC00115 functions as a scaffold lncRNA to link SETDB1 and PLK3 and enhance SETDB1 methylation of PLK3 at both K106 and K200. PLK3 methylation reduces phosphorylation of HIF1α and thereby increases HIF1α stability. HIF1α upregulates ALKBH5 to reduce m^6^A modification of LINC00115, resulting in decreased degradation of YTHDF2-dependent m^6^A-modified RNA and enhanced LINC00115 stability. Thus, this positive feedback loop provokes BCSC phenotypes, contributing to chemoresistance and metastasis. Targeting SETDB1 with a small molecular inhibitor reduces LINC00115-mediated BCSCs stemness and HIF1α expression and enhances BCSCs response to PTX.

To further validate our findings in clinical practice, we examined the expression of LINC00115, SETDB1, PLK3 K106me1, PLK3 K200me1, and HIF1α in metastatic lymph nodes from 118 clinical specimens of TNBC. As shown in Fig. 9a, SETDB1, PLK3-K106me1, PLK3-K200me1, and HIF1α were significantly co-expressed in metastatic lymph nodes with high expression of LINC00115. The statistical significance of these correlations was further supported by the quantification of the IHC and FISH staining (Fig. 9b). Moreover, Kaplan-Meier survival analyses revealed that an increase in expression levels of PLK3 K106me1 or PLK3 K200me1, along with SETDB1 expression, was correlated with a shorter progression-free survival in TNBC patients with metastasis (Fig. 9c and d). Taken together, our data demonstrate that correlative expression of LINC00115, methylation PLK3, SETDB1, and HIF1α are prognostic for clinical TNBC.

## Discussion

The development of therapeutic resistance and metastasis is the main problem in the treatment of breast cancer [47]. Epigenetic regulation, specifically involving non-coding RNA and post-translational modification (PTMs), holds potential as promising diagnostic biomarkers and crucial nodal points for therapeutic intervention in breast cancer therapeutic resistance and metastasis [48, 49]. In this study, we have observed a robust upregulation of LINC00115 in paclitaxel-resistant BCSC. This finding has shed light on a previously unidentified role of LINC00115 in facilitating breast cancer chemotherapy resistance and metastasis. Mechanistically, LINC00115 recruits SETDB1/PLK3 complex to activate the HIF1α signaling pathway. Besides, SETDB1-mediated PLK3 K106 and K200 methylation enhances the phosphorylation of PLK3, leading to the inhibition of its activity. Consequently, the loss of PLK3 activity prevents the phosphorylation of HIF1α and enhances HIF1α protein stability. HIF1α, in turn, upregulates ALKBH5 to reduce m^6^A modification of LINC00115, resulting in hindering the degradation of YTHDF2-dependent m^6^A-modified RNA and thereby stabilizing LINC00115 expression. Ultimately, this positive feedback loop provokes BCSC phenotypes, which contributes to the development of chemotherapy resistance and metastasis (Fig. 9e). Furthermore, inhibiting LINC00115 in combination with SETDB1 inhibitor markedly improved the efficiency of paclitaxel chemotherapy in an animal xenograft model of breast cancer metastasis. Additionally, a concomitant upregulation of LINC00115, SETDB1, PLK3 K106me1, PLK3 K200me1, and HIF1α serves as an indicator of unfavorable prognostic factors for TNBC patients.

In this study, we identified LINC00115 as a new regulator of chemotherapy-resistant BCSC. LINC00115 has been demonstrated to be highly expressed in breast cancer and other cancers and is a poor prognostic factor of cancers [9–14]. Accumulated evidence also demonstrated that LINC00115 functions as a miRNA sponge to regulate cancer stem-like cell stemness and progression [13–15]. Here, we reveal that LINC00115 is upregulated in chemoresistant BCSC and high LINC00115 expression relates to a poor prognosis for breast cancer patients after chemotherapy. In addition, LINC00115 plays a new function as an RNA linker to recruit SETDB1 and PLK3, which facilitates SETDB1 methylating of PLK3 to activate HIF1 signaling and enhance BSCS properties.

We also demonstrate that SETDB1 methylates a new non-histone protein PLK3. SETDB1 is a classical histone methyltransferase to methylate H3K9 [20, 21]. Recently SETDB1 was also shown to methylate several proteins, such as AKT protein kinase [22, 24]. These methylations either augment or impede the respective kinase activities to regulate the development of cancer [23]. In this study, we indicate that SETDB1 binds to and methylates PLK3, which enhances PLK3 phosphorylation but inhibits PLK3 activity to phosphorylate its substrate HIF1α. Consistent with the previous report that SETDB1 is an oncogene in breast cancer and plays an important role in treating endocrine therapy resistance [33, 50–52], our study reveals that SETDB1 is critical for BCSC properties, chemotherapy resistance, and metastasis.

The regulation of HIF1α protein levels and its activity was mediated by a signaling cascade comprising protein kinases and protein/lipid phosphatases [17]. In this study, we discover that methylation of PLK3 increases its phosphorylation but diminishes its kinase activity to phosphorylate HIF1α. In line with this finding, it was reported that PLK3 phosphorylation blocks its kinase activity and prevents the phosphorylation of caspase-8 [39]. In addition, GSK3β phosphorylates HIF1α, leading to an increased degradation of HIF1α through the proteasomal pathway [18]. PLK3 also directly interacts with and phosphorylates HIF1α to promote HIF1α destabilization like that regulated by GSK3β [18, 19]. Our study indicates that the methylation of PLK3 results in the failure to phosphorylate HIF1α, thereby promoting HIF1α protein stability through the inhibition of its ubiquitination degradation pathway. Our study also supports that the upregulation of HIF1α contributes to the enhancement of BCSC properties, resulting in therapy resistance and metastasis.

*N*^6^-methyladenosine (m^6^A) is the most prevalent internal chemical modification of mRNAs and lncRNA in eukaryotes [53, 54], which is widely involved in mRNA or lncRNA metabolism, affecting RNA stability and splicing, RNA nucleation, RNA-protein interaction, and mRNA translation [55, 56]. Aberrant m^6^A RNA methylation has been linked to several diseases including cancers [42, 57]. In this study, we indicate that LINC00115 is modified by m^6^A, which is recognized by ALKBH5 and YTHDF2. ALKBH5 and YTHDF2 are antagonistic to each other in sustaining LINC00115 stability. Moreover, previous research has indicated that ALKBH5 plays a role in facilitating the hypoxia-induced and HIF1α-dependent BCSC phenotype [30]. In our current study, we show that the LINC00115/SETDB1/PLK3 axis-mediated HIF1α increases ALKBH5 expression, which, in turn, enhances LINC00115 stability through demethylation of its m^6^A. This feedback loop further enhances BCSC properties and metastasis.

## Conclusion

In this study, we identify LINC00115 as a new epigenetic regulator of drug-resistant BCSC, which activates the HIF1α signaling through both SETDB1/PLK3 and ALKBH5/YTHDF2 complexes. This study also reveals that SETDB1-mediated PLK3 K106/200 methylation not only serves as a key signal driving HIF1α stability and BCSC properties but also acts as a prognostic factor for metastatic breast cancer patients. Thus, targeting LINC00115 signaling factors may be a potential therapy strategy for patients with therapeutic-resistant breast cancer.

### Electronic supplementary material

Below is the link to the electronic supplementary material.

Supplementary Material 1

## Data Availability

No datasets were generated or analysed during the current study.

## Abbreviations

LncRNAs: Long non-coding RNAs
BCSCs: Breast cancer stem-like cells
TNBC: Triple negative breast cancer
PTX: Paclitaxel
RIP: RNA immunoprecipitation
PLK3: Polo Like Kinase 3
SETDB1 SET: domain bifurcated histone lysine methyltransferase 1
MS: Mass spectrometry
FACS: Fluorescence-activated cell sorting
ELDA: Mammosphere formation and extreme limiting dilution assay
shRNA: Short hairpin RNA
sgRNA: Single-guide RNA
ASO: Antisense oligonucleotide
m6A: N6-Methyladenosine
meRIP: Methylated RNA immunoprecipitation
FISH: RNA fluorescence in situ hybridization
HIF1: Hypoxia-inducible factor 1
ALKBH5: α-ketoglutarate-dependent dioxygenase alkB homolog 5
