# Supplementary material for: LINC00115 promotes chemoresistant breast cancer stem-like cell stemness and metastasis through SETDB1/PLK3/HIF1α signaling
Source: Mol Cancer. 2024 Mar 22;23:60. doi: 10.1186/s12943-024-01975-3 (PMC10958889; doi:10.1186/s12943-024-01975-3)
Supplement: Supplementary file 1 — Supplementary Material 1 [file 12943_2024_1975_MOESM1_ESM.docx]

**LINC00115 promotes chemoresistant breast cancer stem-like cell stemness and metastasis through SETDB1/PLK3/HIF1α signaling**

Fei Luo, Mingda Zhang, Bowen Sun, Chenxin Xu, Yi Yang, Yingwen Zhang, Shanshan Li, Guoyu Chen, Yanxin Li, and Haizhong Feng

Table S1-S2

Figure S1-S2

**Supplementary Table 1** Mass spectrometry analysis of the proteins pulled down by biotinylated LINC00115 in MDA-MB-231 BCSCs.

| Accession | Gene names | MW [kDa] | Score Mascot | Coverage (%) | Unique Peptides | Peptides |
| --- | --- | --- | --- | --- | --- | --- |
| P49327 | FASN | 273.3 | 1810 | 41.8160096 | 77 | 78 |
| P42704 | LRPPRC | 157.8 | 2069 | 51.2912482 | 68 | 68 |
| Q9H4B4 | PLK3 | 71.6 | 2221 | 73.6051502 | 47 | 48 |
| Q15047 | SETDB1 | 143.1 | 1703 | 63.0344828 | 42 | 42 |
| P10809 | HSPD1 | 61 | 2648 | 69.1099476 | 39 | 39 |
| P78371 | CCT2 | 57.5 | 2101 | 62.8037383 | 35 | 35 |
| P11021 | HSPA5 | 72.3 | 2033 | 48.9296636 | 35 | 37 |
| P38646 | HSPA9 | 73.6 | 2389 | 46.539028 | 33 | 33 |
| P49411 | TUFM | 49.5 | 1988 | 68.5840708 | 30 | 30 |
| P08238 | HSP90AB1 | 83.2 | 1795 | 48.2044199 | 23 | 45 |
| P07900 | HSP90AA1 | 84.6 | 1767 | 43.442623 | 23 | 44 |
| P00338 | LDHA | 36.7 | 1017 | 50.3012048 | 20 | 22 |
| Q6P6C2 | ALKBH5 | 44.2 | 965 | 36.0784314 | 17 | 17 |
| P00367 | GLUD1 | 61.4 | 1993 | 52.3297491 | 9 | 28 |
| P07437 | TUBB | 49.6 | 2615 | 69.1441441 | 5 | 29 |
| Q9Y5A9 | YTHDF2 | 62.3 | 833 | 42.7304965 | 4 | 28 |
| P68371 | TUBB4B | 49.8 | 2441 | 68.0898876 | 1 | 28 |
| Q13885 | TUBB2A | 49.9 | 2437 | 66.2921348 | 1 | 26 |
| Q9BVA1 | TUBB2B | 49.9 | 2420 | 66.2921348 | 1 | 26 |

**Supplementary Table 2** Primers for qRT-PCR and meRIP-qPCR assays.

| Primer pairs | Sequence |
| --- | --- |
| qRT-PCR, GAPDH | 5’-GGAGCGAGATCCCTCCAAAAT-3’ and  5’-GGCTGTTGTCATACTTCTCATGG-3’ |
| qRT-PCR, LINC00115 | 5’-TTTTTGTGGCCAAACCCAGC-3’ and  5’-ACAGGCTTCGGAGCATTTCC-3’ |
| qRT-PCR, LDHA | 5’-GGTCCTTGGGGAACATGGAG -3’ and  5’-TAGCCCAGGATGTGTAGCCT -3’ |
| qRT-PCR, MDR1 | 5’-CAACTTGCAAGGGGACCAGAG-3’ and  5’-TTCCTCGAGAAACTGCGAAACA-3’ |
| qRT-PCR, HIF-1α | 5’- GGCGCGAACGACAAGAAAAA-3’ and  5’- GTGGCAACTGATGAGCAAGC-3’ |


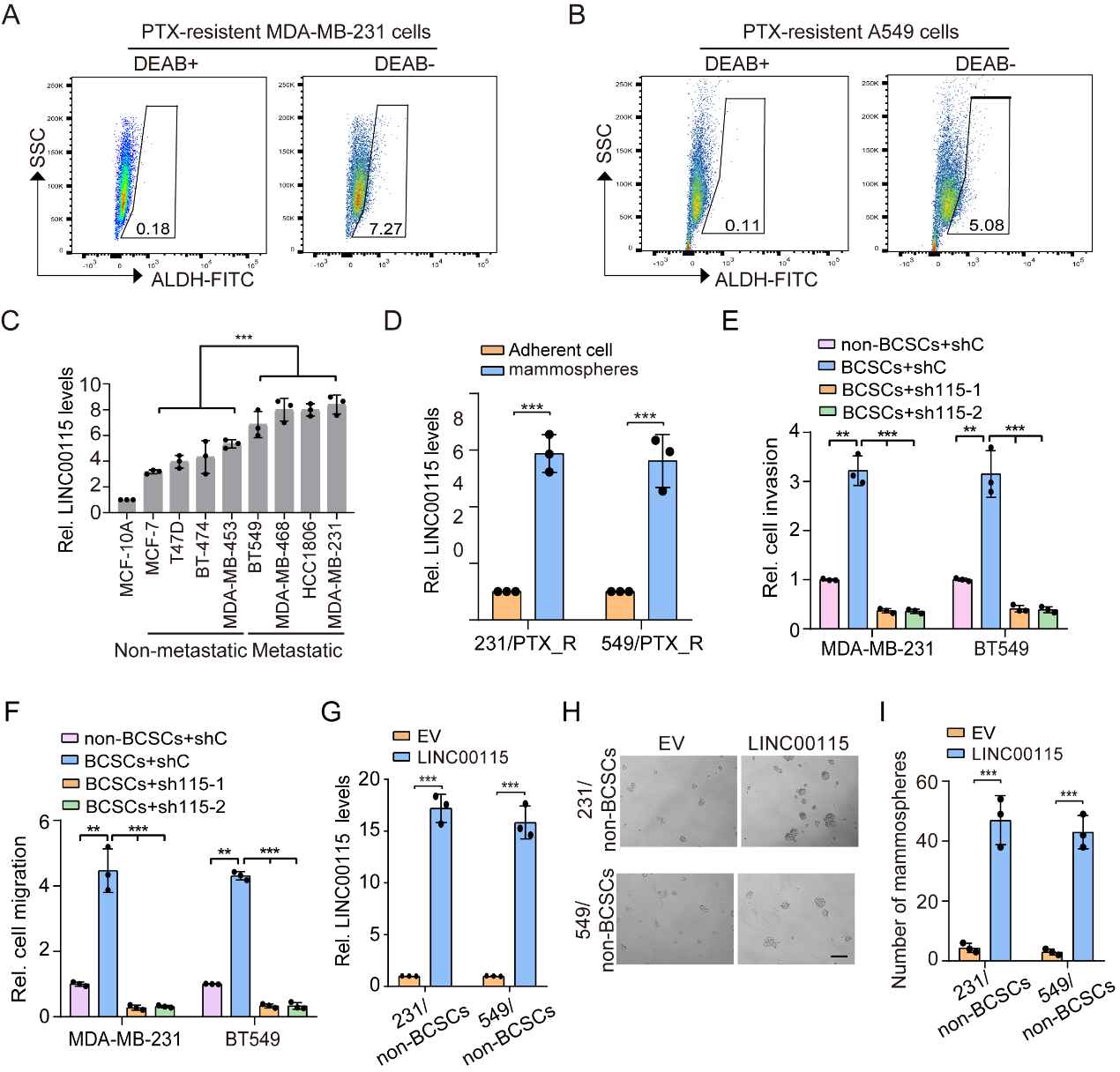


**Supplementary Fig. 1 High LINC00115-expressing TNBC cells had increased stem cell phenotype-forming capacity.**

A-B. Flow cytometry analyses of ALDH+ BCSCs in MDA-MB-231 PTX-resistant (PTX_R) cells and BT549 PTX_R cells.

C. Quantitative real-time RT-PCR (qRT-PCR) analysis of LINC00115 expression in nontumorigenic epithelial cell line MCF-10A, nonmetastatic, and metastatic breast cancer cell lines.

D, The level of LINC00115 was determined in adherent cells and mammospheres from MDA-MB-231/PTX_R and BT549/PTX_R cells.

E-F. Effects of LINC00115 depletion on invasion (E) and migration (F) of paclitaxel-resistant BCSCs and non-BCSCs.

G, QRT-PCR analysis of LINC00115 expression in non-BCSCs derived from paclitaxel-resistant cells transfected with LINC00115.

H-I, Self-renewal ability determined and calculated by mammosphere formation in LINC00115-overexpressed non-BCSCs derived from MDA-MB-231/PTX_R and BT549/PTX_R cells. Scale bars, 50 μm.

Data are representative of three independent experiments with similar results. Data are expressed as the mean ± SD. ***P* <0.01, ****P* <0.001, by two-tailed *t*-test.


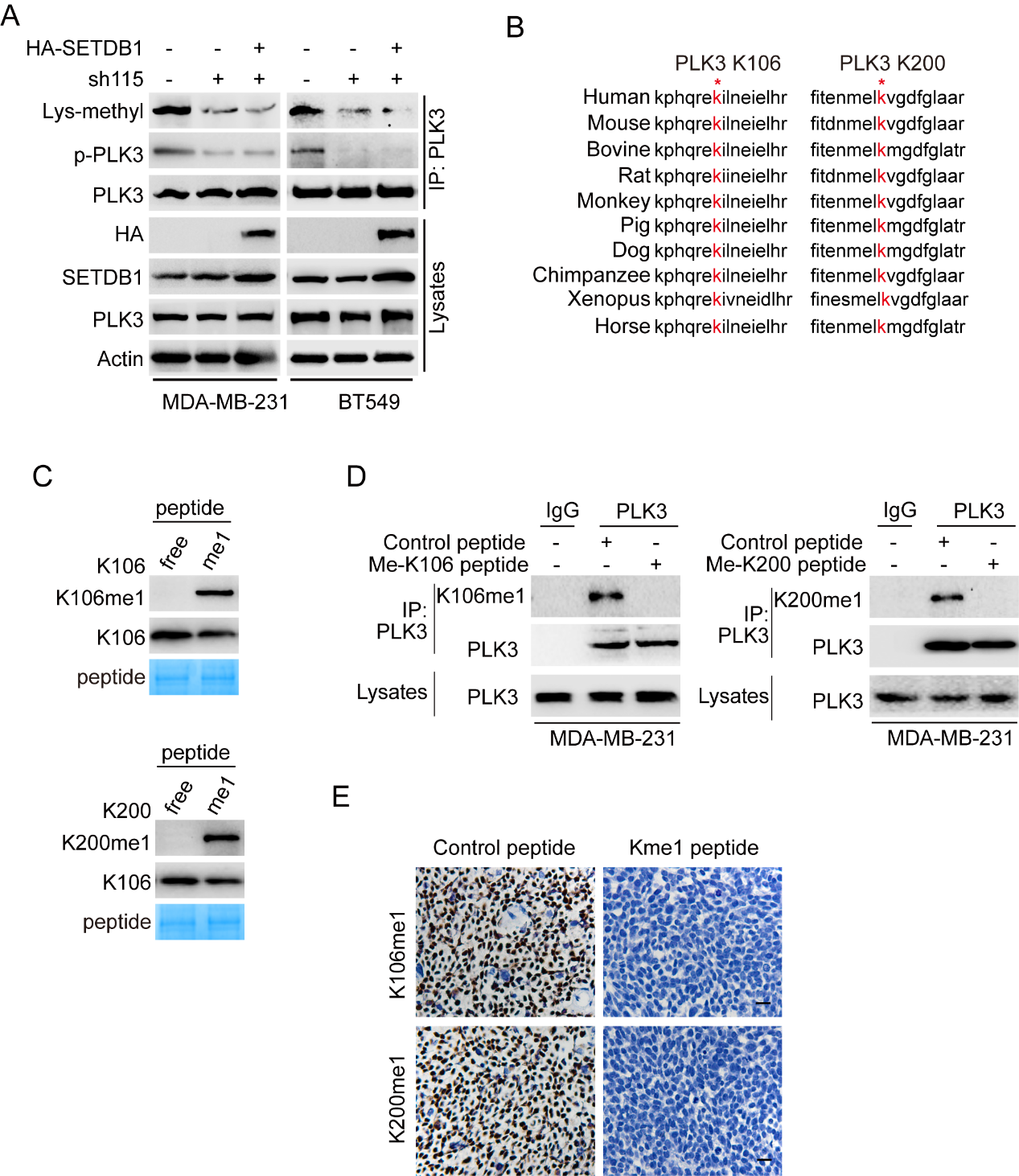


**Supplementary Fig. 2 SETDB1 interacted with and methylated PLK3 at K106 and K200.**

A, IB of the phosphorylation and lysine methylation of PLK3 in control and LINC00115-knockdown MDA-MB-231 and BT549 cells combined with ectopic expression of SETDB1. Lysates were assessed by immunoprecipitation (IP) with anti-PLK3 and immunoblotting with anti-Phospho-(Ser/Thr) and anti-Lys-Mono-Methyl.

B, The amino acid sequences around K106 and K200 in PLK3 among multiple species.

C, IB of PLK3 K106 or K200 mono-methylation antibody with using K106 or K200 unmodified (K106 or K200-free), and K106 or K200 monomethylated (K106 or K200-me1) peptides. Top panel, IB analysis; lower panel, Coomassie brilliant blue staining.

D, IP and WB for PLK3 methylation in MDA-MB-231 cells. Before IP, agarose beads were pre-incubated with a control peptide or the specific methyl-peptide containing me-K106 or me-K200.

E, Immunohistochemistry (IHC) assays of a clinical breast cancer tissue with the specific anti-PLK3-K106me1 and K200me1 antibody in the presence of a control peptide or the specific methyl-peptide containing me-K106 or me-K200. IHC was performed twice on the breast cancer sample with the blocking peptide with similar results. Scale bar, 50 μm.
